# Radiographic, computed tomographic, and histologic characteristics of bone for clinically normal laying hens in a free‐range housing system

**DOI:** 10.1111/vru.13443

**Published:** 2024-10-03

**Authors:** Jeryl Jones, Ahmed Ali, Cerano Harrison, Guillermo Rimoldi

**Affiliations:** ^1^ Department of Animal and Veterinary Sciences Clemson University Clemson South Carolina USA; ^2^ South Carolina Translational Research Improving Musculoskeletal Health Center Clemson University Clemson South Carolina USA; ^3^ Veterinary Diagnostic Center; College of Agriculture, Forestry and Life Sciences Clemson University Clemson South Carolina USA

**Keywords:** Backyard chickens, Egg production, KBD, Keel bone damage

## Abstract

Laying hens are increasingly being kept in backyard flocks and considered family pets; however, diagnostic imaging characteristics of bone for clinically normal backyard hens are currently limited. This prospective, descriptive study was to describe radiographic, computed tomographic, and histologic characteristics of bone for a group of clinically normal laying hens housed in conditions comparable to those of backyard flocks. Sixteen 60‐week‐old Lohmann Brown laying hens were included. Hens were housed in a free‐range unit with outdoor access at a university research and teaching farm. Hens were defined as clinically normal by the farm manager and a veterinary researcher in laying hen behavior and welfare. Findings from the horizontal beam, left lateral, sternal radiographs (*n* = 16), postmortem, and whole‐body CT scans (*n* = 4) were recorded by a veterinary radiologist and a research technician. Histologic findings for sternal, femoral, and tibiotarsal bone samples (*n* = 5) were recorded by a veterinary pathologist. The most frequent radiographic findings for the sternal carina (keel bone) were smoothly marginated concave deviations of the ventral margin and caudal section fractures. Multiple punctate mineral opacities (PMOs) were present in radiographs and CT images for all hens and were involved in the sternal carina and multiple other bones in the axial and appendicular skeleton. No bone abnormalities were identified in any histologic sections where PMOs were radiographically detected. Authors propose that PMOs are normal radiographic and CT findings in the bones of mature, laying hens and may represent temporary calcium reservoirs formed during osteoclastic activities.

## 1 INTRODUCTION

Laying hens are increasingly being kept in backyard flocks and considered as family pets.^1^, ^2^, ^3^, ^4^ Owners of these flocks may request diagnostic imaging consultations when birds develop clinical problems. Multiple previous reports have described radiography and CT as techniques for characterizing sternal carina (keel bone) damage and other measures of bone health in laying hen experimental studies.^5^, ^6^, ^7^, ^8^, ^9^, ^10^, ^11^, ^12^, ^13^, ^14^, ^15^, ^16^, ^17^, ^18^, ^19^, ^20^, ^21^, ^22^, ^23^, ^24^ However, published studies describing diagnostic imaging characteristics of the sternum and other bones for clinically normal, backyard flock, laying hens are currently limited.^25^, ^26^

The sternum plays an important role in avian flight and respiration as muscles responsible for both functions are attached here.^27^, ^28^, ^29^ The sternal carina is a ventral extension of the sternum present in most avian species.^29^ The carina is cartilaginous in origin and ossifies as the bird ages, generally concluding at approximately 40 weeks of age.^30^ Sternal fractures are an important welfare concern in laying hens, and multiple causes have been proposed.^31^ One study reported that 97% of birds had at least one sternal fracture, and 99% had at least one other radiographic finding.^13^ One study described radiographic scores of sternal carina damage for laying hens housed in free‐range conditions; however, no descriptions of other measures or findings for clinically normal birds were found.^20^ One study applied CT segmentation to compare lateral angulation scores and total bone density averages for the sternal carina among conventional cage, free range, and cage‐free housing systems.^18^ Cortical bone thickness values for the tibia and femur at proximal, middle, and distal locations were also compared. However, ranges of CT measures for clinically normal birds in free‐range housing systems were not reported.

The objectives of the current study were to describe radiographic, computed tomographic, and histologic characteristics of bone in a sample of clinically normal, laying hens housed in conditions comparable to those of backyard flocks.

## 2 MATERIALS AND METHODS

### 2.1 Selection and description of subjects

The study was a prospective, descriptive design. Ethical approval and oversight were provided by the Clemson University Institutional Animal Care and Use Committee (protocol number 2021‐020). Sixteen 60‐week‐old Lohmann Brown laying hens were used for the study. This sample size was based on convenience sampling. The group of hens had been used for teaching purposes at our university's research and teaching farm (Clemson University Morgan Poultry Center) and were scheduled for culling as per standard farm procedures. Hens were housed in a free‐range unit (∼4.3 m^2^) with a stocking rate of 16 hens/pen and stocking density of 2150 cm^2^ (1 bird/0.22 m^2^) in addition to outdoor access. The free‐range pen contained adequate nest space, a commercial tube feeder, a water bowl, and 5 cm of wood shavings on the floor as litter. Each pen had approximately 7.6 cm of clean pine wood shavings covering the floor. Feed was given in moveable circular hanging feeders ad libitum, and water was available in automatic cup drinkers ad libitum. Individual nest boxes were provided at the back of each pen (4 hens/nest). The range area was initially 100% covered (before bird access) with a variety of grass and weeds typical to the region, with ∼0.4 m^2^/bird. Each pen contained two round wooden perches (both ∼40 cm from the floor), providing 15 cm perch space per hen. Birds were provided with a commercial diet that followed strain standard guidelines for nutrient requirements (Provimi Corporate Layer 2 with phytase; detailed analyses provided in Supporting Information S1). A single 60‐watt incandescent overhead lightbulb provided light. At 20 weeks, hens were provided with 13.5 h of light, which gradually increased to 16 h at week 30 and then maintained until the end of the study. Hens were observed daily by farm personnel and acclimated to humans through regular interactions with farm staff and students. Hens were defined as clinically normal based on behavioral observations and a consensus between the farm manager and a veterinarian with 15 years of experience as a laying hen behavior and welfare researcher (A.A.). Criteria used for determining normal clinical status were the following: coordinated body movements, normal feathers, no evidence of surface wounds or discharge from body orifices, and normal behaviors such as eating food, drinking water, dust bathing, and foraging.

### 2.2 Data recording and analysis

#### 2.2.1 Radiography of live hens

**FIGURE 1:**
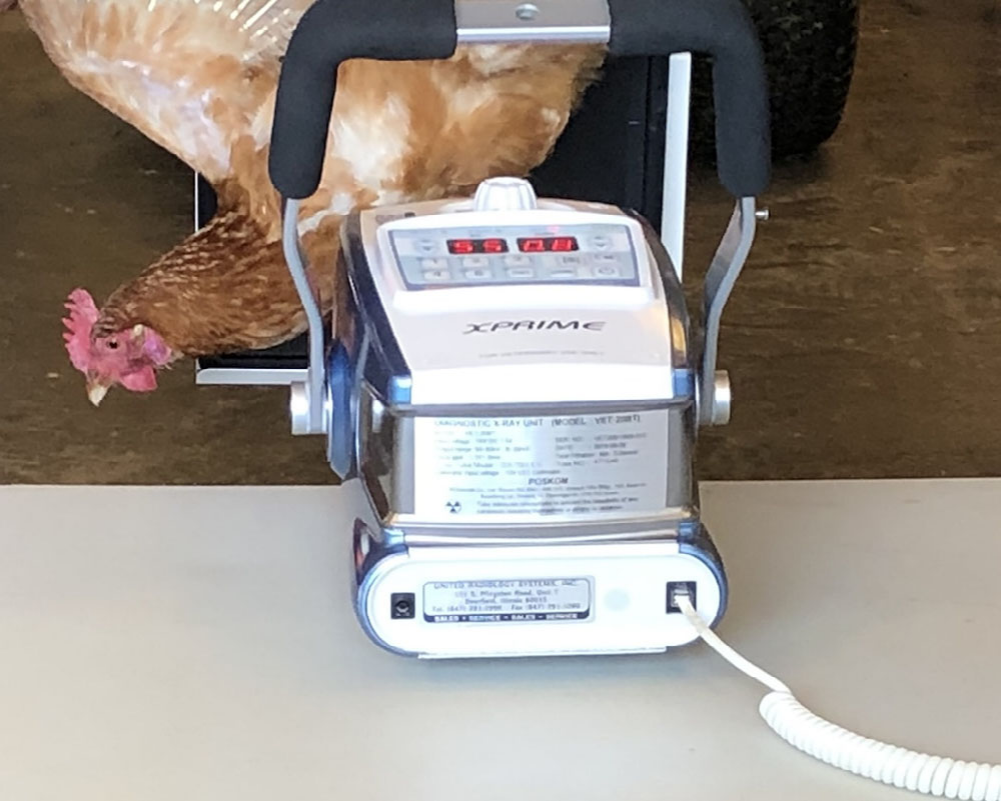
Photograph illustrating horizontal beam positioning used for left lateral radiography of the sternum in an awake laying hen. The portable X‐ray machine was placed on a table and the detector plate was supported using a stand. An experienced veterinarian wearing lead gloves, thyroid shield, and apron carefully suspended the hen upside down next to the plate by the hind limbs and extended the wings dorsally. The collimator was adjusted to include the entire sternum and femur in the field of view.

All radiographic procedures were approved by and conducted in accordance with the requirements of our university's radiation safety officer (Office of Occupational and Environmental Safety, Clemson). All personnel had participated in required training and wore protective lead clothing and personal dosimeters. Radiographs were acquired in the building adjacent to the one where the hens were housed in order to minimize transportation stress. Hens were captured by farm staff and placed in transport crates with a maximum of nine birds/crate. The transport crates were placed on the ground, with butcher paper placed underneath. Experienced staff gently removed hens one at a time from crates by holding both of their feet with the handler's right hand while supporting their breast with the left hand. The laying hen behavior and welfare researcher (A.A.) positioned hens for radiography using a minimal‐stress technique he had previously developed. He suspended hens’ heads down and held them by their hind limbs while gently holding their wings in dorsal extension for horizontal beam, left lateral radiographs of the sternum (Figure 1). Technical staff from our university's animal research center (Godley Snell Research Center, Clemson University) acquired radiographs under the supervision of an ACVR‐certified veterinary radiologist (J.J.). All radiographs were acquired using the same portable radiography machine and technique settings (Sound, Smart DR, 50.0 kV, 2.5 mAs). The DICOM format images for each radiograph were transferred to a PACS and archived. Hens were returned to their pen immediately after radiography and monitored by the laying hen behavior and welfare researcher (A.A.) for signs of clinical problems.

#### 2.2.2 Cadaver specimen sample collection

Two weeks following radiography, all hens were humanely euthanized by cervical dislocation, an AVMA‐endorsed euthanasia procedure for poultry.^32^ Euthanasia was performed by the laying hen behavior and welfare researcher (A.A.), who had extensive training and had previously conducted cervical dislocation for more than 5000 birds. Each hen was assigned a research number, placed in a sealed plastic bag, positioned with wings flexed and hindlimbs extended caudally and placed in a cooler with ice. Five of the 16 hen cadavers were dissected within 2 h of euthanasia for use in histologic examinations. Muscles were removed from the sternal bones, femurs, and tibiotarsal bones. Bones were placed in formalin (10% buffered). The remaining hen cadavers were kept intact and stored in a freezer (−18°C).

#### 2.2.3 Computed tomography of cadaver hens

**FIGURE 2:**
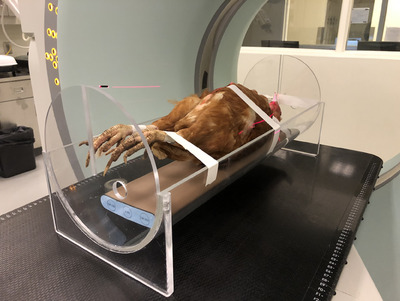
Photograph illustrating positioning used for postmortem, whole‐body CT of a hen. A radiolucent positioning device was placed on the table, and a bone calibration phantom was placed on the floor of the device. The hen was positioned in dorsal recumbency, with the hind limbs extended caudally and the wings tucked close to the sides.

Four of the 11 frozen cadaver hens were thawed in a refrigerator for 24 h and transported to another university's Veterinary Teaching Hospital (University of Georgia College of Veterinary Medicine) for CT. The remaining seven cadavers were used in another study (data not published). Birds were positioned in dorsal recumbency on a bone calibration phantom placed in an acrylic positioning device (VetMousetrap, Universal Medical Systems; Figure 2). Whole‐body scans were acquired by a certified CT technologist, in consultation with the veterinary radiologist (J.J.), using a 64‐slice CT scanner and the hospital's standard protocols for avian species (Siemens Sensation 64, kVP 120, mA 210, 1 mm slice thickness for convolution kernel B40s and 0.6 mm slice thickness for convolution kernel B70s). Scans were archived in the hospital's PACS, and a link for accessing DICOM files was shared with researchers.

#### 2.2.4 Radiography and histology of formalin‐fixed bone specimens

Formalin‐fixed bones from the five dissected hen cadavers were radiographed at the Godley Snell Research Center using the same protocol as that used for live hens. Bones were then returned to the formalin solution and transported to our university's diagnostic laboratory (Veterinary Diagnostic Center, Clemson University) for histology. Bone samples were placed in a commercial decalcification solution (Cal‐Ex II Fixative/Decalcifier, Fisher Chemical) for 7 to 10 days. After 1 week in decalcifier solution, the tissues were tested daily until found suitable for sectioning and embedding. An ACVP‐certified veterinary pathologist (G.R.) selected section locations based on radiographic findings. Sections 3 to 4 mm thick were trimmed, paraffin‐embedded, and processed routinely to produce 4 µm sections that were then stained routinely with hematoxylin and eosin (H&E).

#### 2.2.5 Descriptive analyses

Radiographic and CT images were retrieved and analyzed using a dedicated image analysis workstation and open‐source software (Mac OS High Sierra, 10.13.6, MacPro Quad Core, Apple, Inc, Horos v3.3.6, https://horosproject.org/). The veterinary radiologist (J.J.) recorded qualitative findings for all radiographic and CT studies. A researcher with 4 years of image analysis experience (C.H.) recorded the results of quantitative analyses for radiographic and CT images using previously published standardized protocols^15^, ^24^ (reprinted in Supporting Information 2 and 3). The veterinary pathologist (G.R.) recorded histologic findings. The veterinary radiologist and researcher performed descriptive analyses of recorded findings using spreadsheet software (Excel, Microsoft Corp.)

## 3 RESULTS

### 3.1 Radiographic findings

Positioning and image acquisition for each hen were completed in less than 2 min. Most hens remained calm and tolerated the procedure well. If a hen became agitated, it was returned to the transport crate for a rest period before repeating the procedure. All hens were examined by the laying hen behavior and welfare researcher (A.A.) before and after radiographic procedures and found to be clinically normal. The group of hens demonstrated an average production rate of 93.56% ± 5.85% over the study period, with a mean egg weight of 63.55 ± 1.85 g.

**FIGURE 3:**
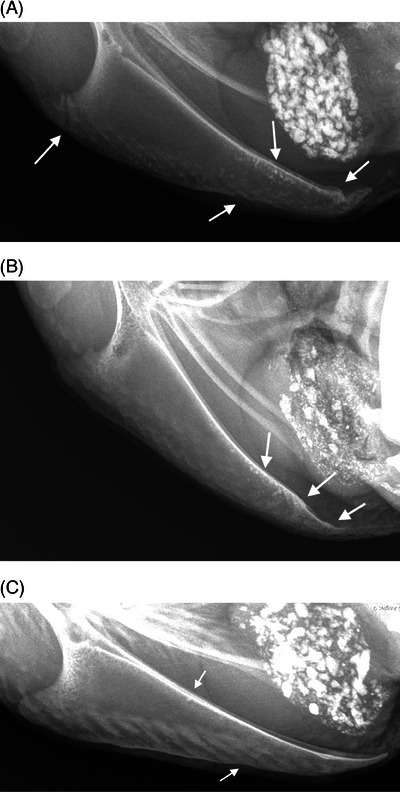
Horizontal beam, left lateral, sternal radiographs illustrating examples of qualitative findings (Sound, Smart DR, 50.0 kV, 2.5 mAs). A, Chronic, nondisplaced fracture of the apex of the carina, PMOs, mild deviation of the ventral margin of the carina, healed fracture of the median trabecula with callus formation and dorsal angulation of the caudal segment. Mineral opacity material in the abdomen is due to gravel in the ventriculus (gizzard). B, Callus formation in the caudal portion of the carina, PMOs, and a nondisplaced complete fracture of the median trabecula. C, Mild deviation of ventral margin, one PMO, no fractures.

Results of qualitative radiographic assessments for the live hens are detailed in Table 1. Results of quantitative radiographic analyses of the sternal carina are detailed in Supporting Information S4. The most frequent findings in the sternal carina were smoothly marginated concave deviations of the ventral margin (Figure 3A) and caudal section fractures (Figure 3B). Fractures were not observed in other skeletal structures included in the radiographs. Multiple, small, discretely marginated, mineral attenuating foci (subsequently termed “punctate mineral opacities or PMOs”) were detected in the sternal carina (Figure 3A–C). These PMOs were also visible in the femurs and tibiotarsal bones for some of the hens. There was a median value of zero for all but one of the quantitative measures of sternal carina damage (Supporting Information S3). The median number of caudal section complete fractures of the sternal carina was two (*n* = 16, range 0–5).

**TABLE 1:** Number and frequency of clinically normal laying hens with qualitative findings in horizontal beam, left lateral, sternal radiographs (*N* = 16).

Sternal carina											Femurs	Tibiotarsal bones
	Remodeling (deviation) of ventral margin	Remodeling (deviation) of dorsal margin	Acute, incomplete fracture	Acute, complete, nondisplaced fracture	Acute, complete, displaced fracture	Acute, comminuted fracture	Chronic, healed fracture	Chronic, nonunion fracture	Chronic, malunion fracture	Punctate mineral opacities	Punctate mineral opacities	Punctate mineral opacities
Cranial	12 (75%)	11 (69%)	13 (81%)	0 (0%)	0 (0%)	0 (0%)	1 (6.3%)	3 (19%)	0 (0%)	2 (13%)	4 (25%)	1 (6.3%)
Middle	14 (88%)	0 (0%)	16 (100%)	2 (13%)	0 (0%)	0 (0%)	0 (0%)	0 (0%)	0 (0%)	9 (56%)		
Caudal	13 (81%)	13(81%)	11 (69%)	7 (44%)	1(6.3%	0 (0%)	5 (31%)	2 (13%)	9 (56%)	6 (38%)		

### 3.2 CT findings

**FIGURE 4:**
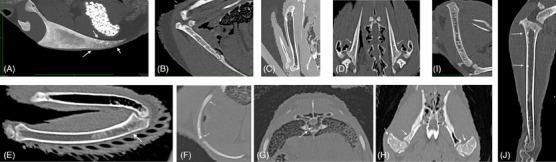
Postmortem, oblique MPR CT images illustrating PMOs in multiple bones (Siemens Sensation 64, kVP 120, mA 210, 0.6 mm slice thickness, convolution kernel B70s, standard bone WW/WL display setting). A, Sternum, mid‐sagittal, thick slab image. Multiple, nondisplaced, complete fractures are also visible in the caudal portion of the carina and median trabecula. There is also mild ventral margin deviation. B, Coracoid bone, mid‐sagittal. C, Humerus, mid‐sagittal. D, Scapulae, dorsal planar. E, Ulna, mid‐sagittal. F, Rib, mid‐sagittal. G, Thoracic vertebra, transverse. H, Pelvis and femoral heads, dorsal planar. I, Femur, midsagittal. J, Tibiotarsus, midsagittal.

**FIGURE 5:**
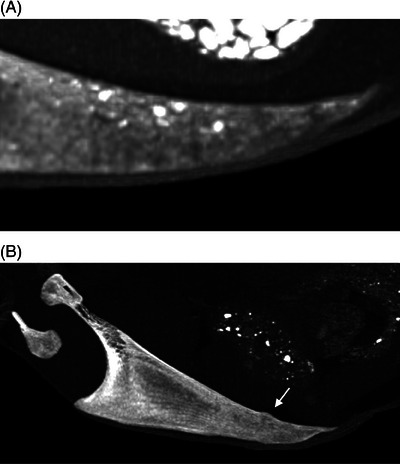
Postmortem, oblique MPR CT images of the sternum illustrating examples of fractures and callus formation. A, Zoomed, mid‐sagittal, thick slab image of the same hen as Figure 4A, illustrating nondisplaced, complete fractures of the caudal section of the carina and median trabecula, with PMOs. B, Mid‐sagittal, thick slab image illustrating callus formation in the caudal section of the carina.

**FIGURE 6:**
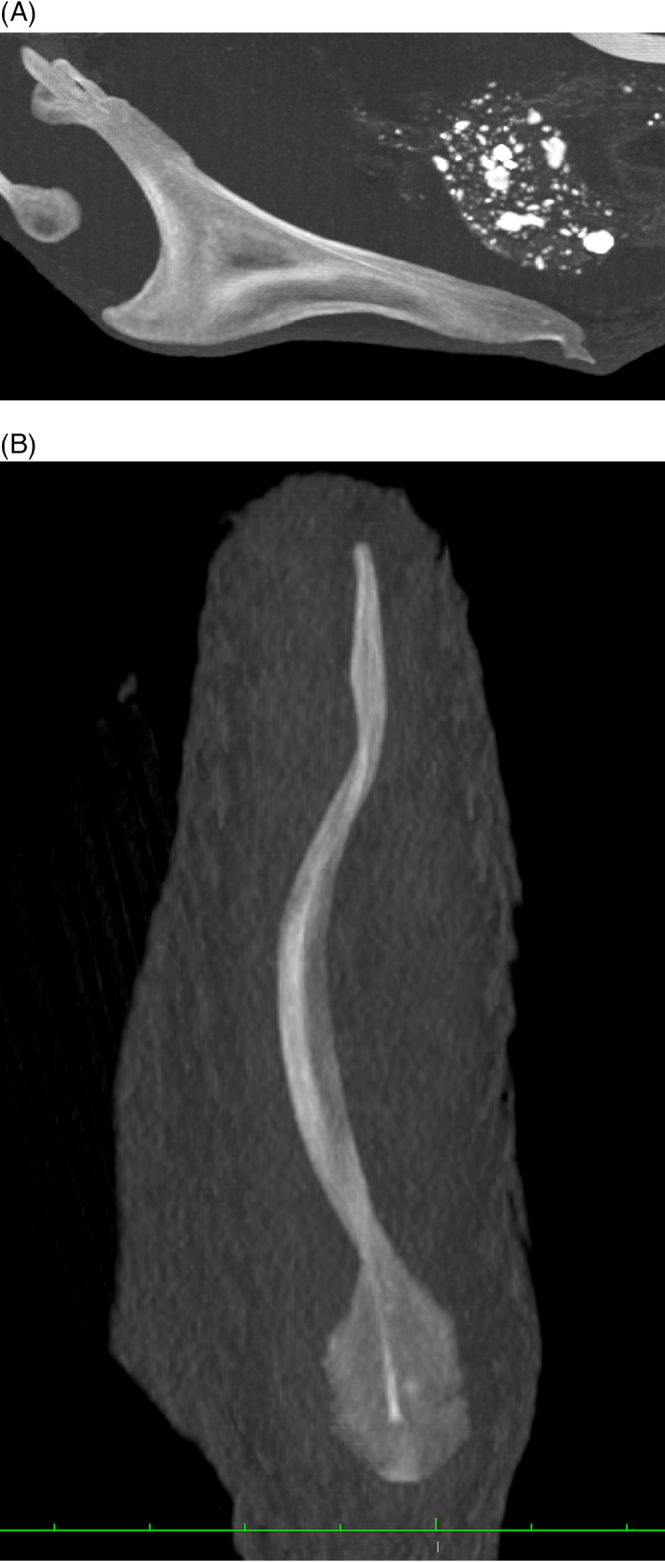
Postmortem, oblique MPR CT images of the sternum illustrating examples of deviation and lateral angulation of the ventral margin of the carina. A, Mid‐sagittal, thick slab image illustrating marked ventral margin deviation and angulation of the median trabecula both ventrally and dorsally. A single PMO is noted. B, Dorsal planar thick slab image illustrating lateral angulation of the ventral carina in cranial, middle, and caudal portions. PMOs are also noted in the caudal portion.

**FIGURE 7:**
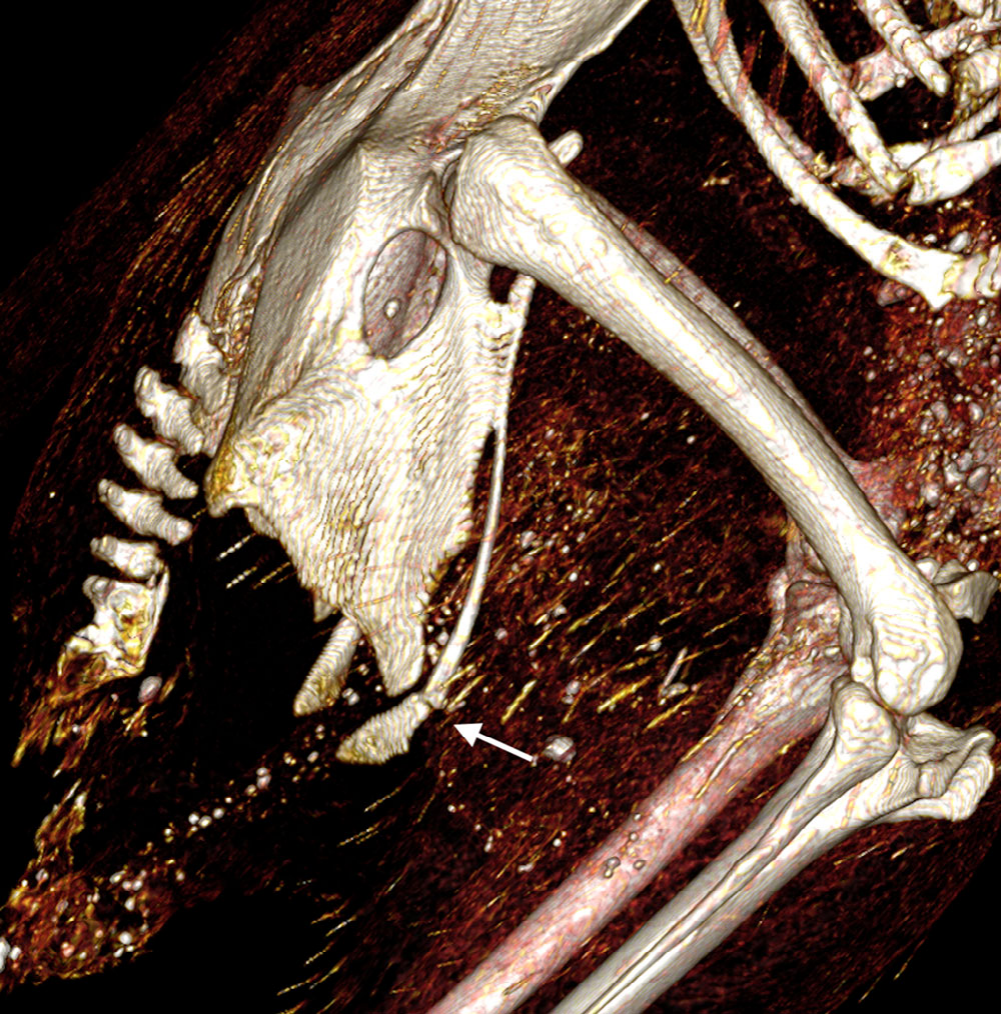
Postmortem, 3D, volume‐rendered CT image illustrating a minimally displaced fracture of the right pubic bone. Callus formation is also noted in the caudal sternal carina.

The results of qualitative CT analyses are detailed in Table 2. The results of quantitative CT analyses are detailed in Supporting Information S5. Calcified eggs were visible in the caudal abdominal cavity for three of the four hens. The most frequently detected qualitative CT findings were PMOs. These were present in all four hens and involved nearly all bones of the axial and appendicular skeleton (Figure 4A–J). These PMOs were located near the junction of cortical and medullary bone, within the medullary bone, or within the trabecular bone. Fractures were detected in the caudal section of the sternal carina for two hens (Figure 5A), and callus formation was detected in the caudal section of the sternal carina for one hen (Figure 5B). Concave deviations and lateral angulations of the sternal carina ventral margin were identified in three hens (Figure 6A, B). One hen had a minimally displaced fracture of the caudal portion of the pubic bone (Figure 7). No other fractures were identified. The tibiotarsal total bone area was subjectively largest in the proximal region (median 55.7 mm^2^). Tibiotarsal total bone mineral density was subjectively largest in the middle region (median 877.5 mg/cm^3^ CaHA). The tibiotarsal cortical bone area was subjectively largest in the proximal region (median 28.5 mm^2^). Tibiotarsal cortical bone mineral density was subjectively largest in the distal region (median 790.6 mg/cm^3^ CaHA). The tibiotarsal muscle area was subjectively largest in the proximal region (median 10.5 cm^2^). Tibiotarsal muscle corrected mean density values were subjectively largest in the distal region (median 291.6 HU). All sternal bones were 100% ossified. The sternal carina exhibited lateral angulation of the ventral margin ranging from 0° to 28° (median 22.9°).

**TABLE 2:** Number and frequency of clinically normal laying hens with qualitative findings in postmortem, whole body computed tomography images (*N* = 4).

Anatomic region	Fractures	Punctate mineral opacities	Callus formation	Bone margin deviation or angulation
Spine	0 (0%)	2 (50%)	0 (0%)	0 (0%)
Furcula	0 (0%)	0 (0%)	0 (0%)	0 (0%)
Coracoid	0 (0%)	3 (75%)	0 (0%)	0 (0%)
Scapula	0 (0%)	3 (75%)	0 (0%)	0 (0%)
Ribs	0 (0%)	2 (50%)	0 (0%)	0 (0%)
Sternum	2 (50%)	2 (50%)	1 (25%)	3 (75%)
Pelvis	1 (25%)	4 (100%)	0 (0%)	0 (0%)
Humerus	0 (0%)	4 (100%)	0 (0%)	0 (0%)
Ulna	0 (0%)	4 (100%)	0 (0%)	0 (0%)
Radius	0 (0%)	0 (0%)	0 (0%)	0 (0%)
Femur	0 (0%)	4 (100%)	0 (0%)	0 (0%)
Tibiotarsus	0 (0%)	4 (100%)	0 (0%)	0 (0%)

*Note*: Calcified eggs were present in three hens.

### 3.3 Postmortem radiographic and histologic findings

**FIGURE 8:**
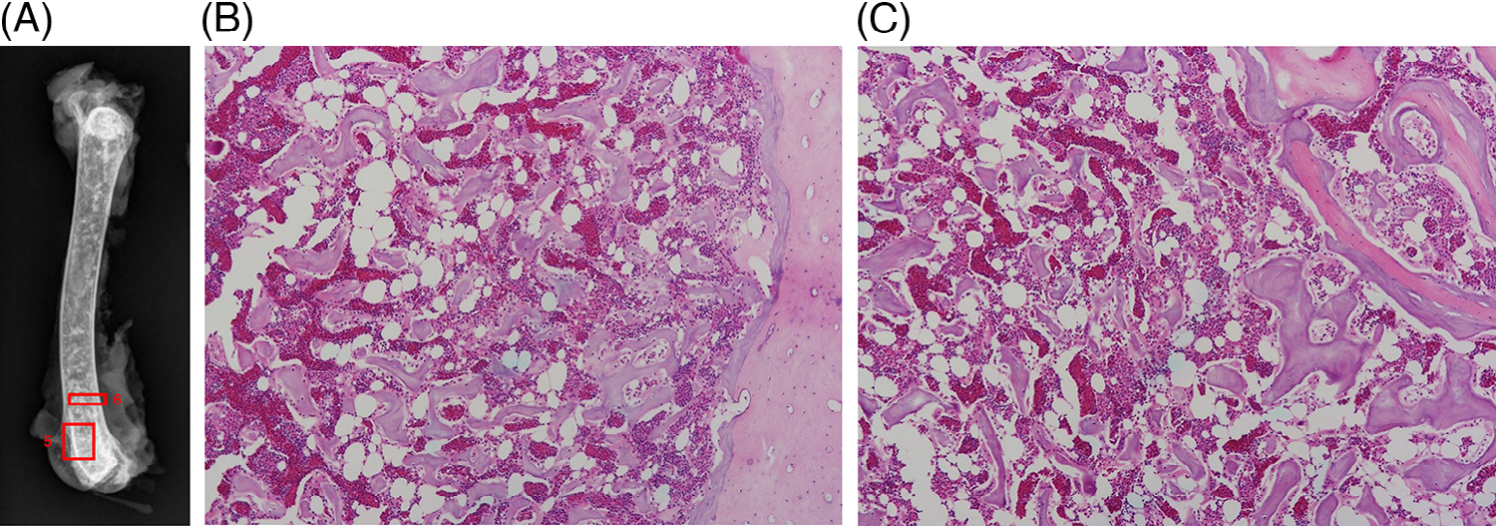
A, Postmortem radiographic image illustrating PMOs in the femur and how locations for histologic sectioning were selected. B, Histologic image for location numbered “5” that included a large concentration of PMOs in the trabecular bone of the distal femoral metaphysis. Femoral cross‐section, note normal cortical bone to the right and normal trabecular bone with bone marrow to the left. C, Histologic image for the location numbered “6” that included a large concentration of PMOs in the cortical and medullary bone of the distal femoral diaphysis. Femoral longitudinal section, note normal cortical bone in the top right corner, and normal trabecular bone with bone marrow to the bottom left. All bone structures were interpreted to be normal at these locations and no explanation for the PMOs was found. (H&E,100× magnification).

Sternal bones for two of the hens were used for another study (data not published), so the sample for these analyses included three sternal bones, five femurs, and five tibiotarsal bones. Radiographs of all dissected, formalin‐fixed bones for the five sampled birds demonstrated multiple PMOs. Chronic caudal section fractures were noted in all three sternal bones. Locations for histologic sections were selected based on the greatest number of PMOs detected on radiographs. For femurs and tibiotarsal bones, sections of the metaphysis (longitudinal) and diaphysis (transverse) were obtained approximately 1 cm away from the metaphyseal section (Figure 8A). A total of 28 sections were examined. On microscopic examination, no significant changes were detected in the locations of the PMOs. The reason for the PMOs was not identified in any of the sections examined. There was no evidence of focal sclerosis, enostoses, or thickening of trabecular bone (Figure 8B, C). Trabecular bone, medullary bone, and cortical bone sections were interpreted to be normal for all sections.

## 4 DISCUSSION

This study intended to provide background information used by veterinarians, veterinary radiologists, and poultry researchers when evaluating radiographs and CT scans of backyard flock laying hens. This group of birds was a strain commonly used in backyard flocks and was in the late laying phase of their life cycle at the time of this study. All birds were carefully examined by experts in laying hen behaviors and determined to be clinically normal. None of the hens had evidence of comminuted fractures or displaced, complete fractures involving the middle or cranial sections of the sternal carina that were described in previous reports. Quantitative radiographic and CT analyses yielded median values of zero for all variables except complete fractures of the caudal section of the sternal carina. We identified PMOs in radiographs and CT images for all hens, and these involved the sternal carina and multiple other bones in the axial and appendicular skeleton. An experienced veterinary pathologist examined 28 sections of formalin‐fixed bone selected from regions with radiographic evidence of PMOs and no evidence of bone pathology was seen in these locations.

Previous publications have described recumbent techniques for acquiring sternal radiographs in chickens.^5^, ^6^, ^25^, ^26^ We chose to acquire sternal radiographs using a head down, left lateral, and horizontal beam technique based on recommendations by the laying hen behavior researcher and his previous experience applying this as a minimal stress technique. The main advantage of this technique was speed, with positioning and acquisition times averaging less than 2 min. Disadvantages of this technique were that hens had to be hand‐held and only lateral views were available for radiographic analyses.

Bone quality is a welfare concern in laying hens because the process of egg laying is calcium‐intensive, with 10% of the hens' body calcium being used to produce an eggshell.^33^ This roughly equates to 2–3 g of calcium per egg. With strains of modern‐day layers laying in excess of 300 eggs per year, the body is in a constant state of bone turnover. At sexual maturity, laying hens develop medullary bone, a metabolically active bone type that serves as the main source of calcium for egg production.^34^, ^35^ During inactive stages of eggshell production (daytime), osteoblastic activity deposits medullary bone on the inner cortical surfaces of bones. At the onset of eggshell formation (nighttime), calcium in the blood is first used to develop the eggshell. With the available calcium in the blood being around 25 mg and calcium deposition occurring at a rate of around 125 mg/h, another source of calcium is needed to form the shell.^33^ Depleted serum calcium levels stimulate parathyroid hormone release, which signals osteoclasts to act selectively on medullary bone. The medullary bone, once resorbed by osteoclastic activity, frees the calcium from hydroxyapatite, and the calcium is released into the blood to further contribute to building the eggshell. If there is an insufficient amount of medullary bone present in the body, osteoclasts then begin to act on cortical bone. This results in thinning cortices, and if prolonged demineralization of cortical bone occurs, the hens develop osteoporosis and become more susceptible to sustaining potentially painful fractures in their housing systems.^31^

Sternal carina ventral margin deviation and lateral angulation findings in our sample were consistent with previous reports.^36^, ^37^, ^38^, ^39^ These chronic, inactive bone margin changes have been attributed to perching behaviors. Perching is an antipredatory behavior exhibited by laying hens in which the birds roost on an elevated surface. In nature, jungle fowl and feral domestic hens can be observed perching in trees, but in captivity, this occurs on perches placed in their housing systems or on other structures positioned off the floor in their enclosures.^40^ While perching, pressure applied to the ventral aspect of the sternal carina during ossification has been proposed to change the shape of the bone over time. The natural, convex curvature of the ventral margin becomes indented at the location where the chest rests on the perch. However, unlike previously published studies, the severity of sternal damage in our sample of birds was low. The median numbers of sternal fractures for our group of hens were predominantly zero, and no other bone fractures (with the exception of one pubic bone fracture) were found based on whole‐body CT evaluations for four of the birds. Possible reasons why hens in our study may have had less severe sternal damage than those reported in previous studies include the following: smaller sample population, different housing system, different bird ages and genetics, higher activity levels, more time together for developing pecking orders and therefore fewer fights over perches, food, nest boxes, etc.; more human interactions so more close monitoring and fewer episodes of being startled. All hens in our study exhibited normal behaviors based on careful assessments by experienced observers. However, the authors acknowledge that the possibility of subclinical pain cannot be completely ruled out. As with many other avian species, laying hens may mask clinical signs of pain.

Authors chose the term “punctate mineral opacities or PMOs” based on standard veterinary radiology roentgen sign terminology. The cause of PMOs observed in radiographic and CT images remains uncertain in spite of careful histologic examinations of multiple bone locations where they were visible. Based on the anatomic locations of PMOs and the absence of bone pathology in these locations, authors hypothesize that PMOs were most likely caused by temporary calcium reservoirs that were formed during osteoclastic activity and subsequently cleared during decalcification tissue processing procedures. One previous publication described “radiopaque spots” in a figure legend illustrating a laying hen CT image of the sternum.^38^ A second previous publication described “medullary calcium chunks” in a figure legend illustrating micro‐CT images of tibiotarsal bones.^41^ A third previous publication included a brief statement that polyostotic hyperostosis could sometimes appear as “discrete mineral opacities resembling osteomas” in birds; however, the referenced figure illustrated a diffuse increase in medullary opacity.^42^ A fourth previous publication noted polyostotic hyperostosis as a normal finding in a figure legend, also illustrating a diffuse increase in medullary bone opacity for a young hen.^25^ A previous review article described an imaging finding termed “spotted bones” in humans.^43^ One cause was termed “osteopoikilosis” and this was described as a benign, incidental finding of unknown cause. None of these publications described the histologic characteristics of these findings. Other previous studies have reported histologic features of bones in laying hens; however, no descriptions of causes for PMOs were found.^33^, ^35^, ^44^, ^45^, ^46^, ^47^ One study described histologic changes that occurred in bone during the onset of lay (sexual maturity).^35^ At 12 weeks of age, cavities were observed in the cortical bone, which was filled with medullary bone. Two reports described histologic characteristics of cortical bone resorption at the junction of cortical and medullary bone and found that medullary bone replaced the lost cortical bone material.^33^, ^44^ One study described histologic evidence that sternal carina damage corresponded with callus formation but found that not all damage, such as deviations/deformities, resulted in the development of callus material.^45^ One study reported histologic evidence that calluses arose from the periosteum.^46^ One study reported histologic evidence of a reduction in trabecular bone volume in response to reduced activity.^47^

Limitations of the current study were that we performed postmortem CT, postmortem radiography, and histologic examinations for a subsample of the hens rather than for all of them. In addition, we performed histology only using decalcified specimens of the sternum, tibiotarsus, and femur and routine staining. The birds used for CT were also not the same as the birds used for histology. These choices were made based on financial constraints and scheduling constraints for CT scanning at another university. Another limitation of the study was the small sample size that precluded statistical comparisons.

In conclusion, the current study introduced qualitative and quantitative radiographic and CT characteristics of bone in a sample of clinically normal Lohmann Brown hens that were in the late laying phase of their life cycle and were housed in free‐range conditions comparable to those used for backyard flocks. We also introduced radiographic and CT characteristics of findings termed “punctate mineral opacities or PMOs” that were present in multiple bones of the appendicular and axial skeleton. Sternal bones, tibiotarsi, and femurs appeared histologically normal in the locations where the PMOs were detected radiographically. Authors propose that PMOs may be normal radiographic and CT findings in the bones of mature, laying hens and may represent temporary calcium reservoirs formed during osteoclastic activities. It was also considered that the PMOs might have been calcium deposits that were artifactually dissolved during the decalcification process, and the possibility of future studies working with microscopic examination of nondecalcified tissue sections might provide further useful information. Studies comparing numbers of PMOs with genetic factors and gold standard measures of osteoclastic activity may also be helpful in determining whether they could be used as biomarkers in future research studies. Researchers who use total bone density as a measure of intervention effects for experimental studies may want to consider PMOs as possible outside sources of variation in their statistical models.

## CONFLICT OF INTEREST STATEMENT

The authors declare no conflict of interest.

## PREVIOUS PRESENTATION OR PUBLICATION DISCLOSURE

None.

## REPORTING CHECKLIST DISCLOSURE

STROBE Vet guidelines were used.

## Supporting information



Supporting Information

Supporting Information

Supporting Information

Supporting Information

Supporting Information

## Data Availability

Data are available from the corresponding author upon reasonable request.
