# Supplementary material for: Radiographic, computed tomographic, and histologic characteristics of bone for clinically normal laying hens in a free‐range housing system
Source: Vet Radiol Ultrasound. 2024 Oct 3;66(1):e13443. doi: 10.1111/vru.13443 (PMC11617609; doi:10.1111/vru.13443)
Supplement: Supplementary file 1 — Supporting Information [file VRU-66-0-s002.pdf]

Supplement 1. Ingredient percentage and calculated nutrient analysis of peak lay diet used for the 16 sampled hens.

**Ingredient**	**(%)**
Corn	55.03
SBM	28.11
Calcium carbonate	9.09
Soybean oil	2.66
Wheat middlings	2.00
Mono-dicalcium phosphorus	1.41
Sodium chloride	0.44
Choline chloride	0.36
DL-Methionine	0.34
L-Threonine	0.12
Valine	0.10
Isoleucine	0.10
Vitamin/Mineral Premix1	0.05
**Calculated analysis**	
Crude protein	17.82
Crude fat	3.96
Crude fiber	2.18
Calcium	3.87
Phosphorus	0.67
**Metabolizable Energy**	**(kcal/kg)**
	2843.93

Notes: Samples of all diets were analyzed to confirm nutrient composition. Diet was composed of selenium 255 ppm, zinc 6.5%, vitamin A 8294000 IU/kg, phytase activity 399166.2 FTU/kg.
