# Supplementary material for: Radiographic, computed tomographic, and histologic characteristics of bone for clinically normal laying hens in a free‐range housing system
Source: Vet Radiol Ultrasound. 2024 Oct 3;66(1):e13443. doi: 10.1111/vru.13443 (PMC11617609; doi:10.1111/vru.13443)
Supplement: Supplementary file 2 — Supporting Information [file VRU-66-0-s005.pdf]

Supplement 1: Detailed, step-by-step protocol for quantifying radiographic KBD in laying hens[a]

1.) Upon entering image analysis laboratory, turn off main overhead lights and turn on side lamp.
2.) Power on workstation & login using the appropriate logon credentials
3.) Launch Horos software
4.) After Horos has successfully launched, select study of interest from the database list. Double click to open the study.
5.) Once opened, adjust the mouse functions for the left/right button and the roller
    - Left: Density adjustment
    - Right: Zoom
    - Roller: Reposition image
        - To utilize each of these functions, the button must be depressed before moving the mouse to make the adjustment

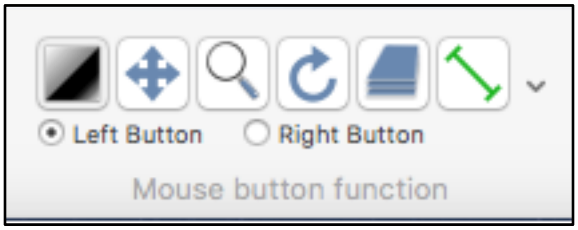

6.) Set the Window level/Window width (WL/WW) to bone.
7.) To randomize the order of studies to be interpreted, go to random.org
    a. Select numbers, then sequences, then insert 1 as the smallest value and set the largest value based on the total number of images to be analyzed to randomize the sequence, then click get sequence
    b. If triplicate measures are needed, create 3 random lists and save them for future reference
8.) Alternatively, open Microsoft Excel

---

[a] Reprinted from previous open access publication. Harrison C, Jones J, Bridges W, Ali A. Intra-observer repeatability for a standardized protocol to quantify keel bone damage in laying hens using discrete and continuous radiographic measures. *Veterinary Radiology & Ultrasound*. 2023.

a. In a column, populate a series of cells in numerical order (1-X, X being the total number of images)

b. In the cell directly to the cell containing the value of "1", enter the formula "=RAND()"

	A	B
1	1	=RAND()
2	2	
3	3	
4	4	
5	5	

c. Copy this formula to the subsequent cells up to the "X" value utilizing the click and drag method. This will create **non-repeating** random numbers for each cell that contains the formula

	A	B
1	1	0.35278826
2	2	0.35362208
3	3	0.85206879
4	4	0.00289552
5	5	0.05839963

d. Then, select all cells, both 1-X & randomly generated numbers, and under the "Data" tab, sort the numbers using "Sort"

e. You will be prompted to sort by one of the columns. Select the column containing the random numbers (column B in this example).

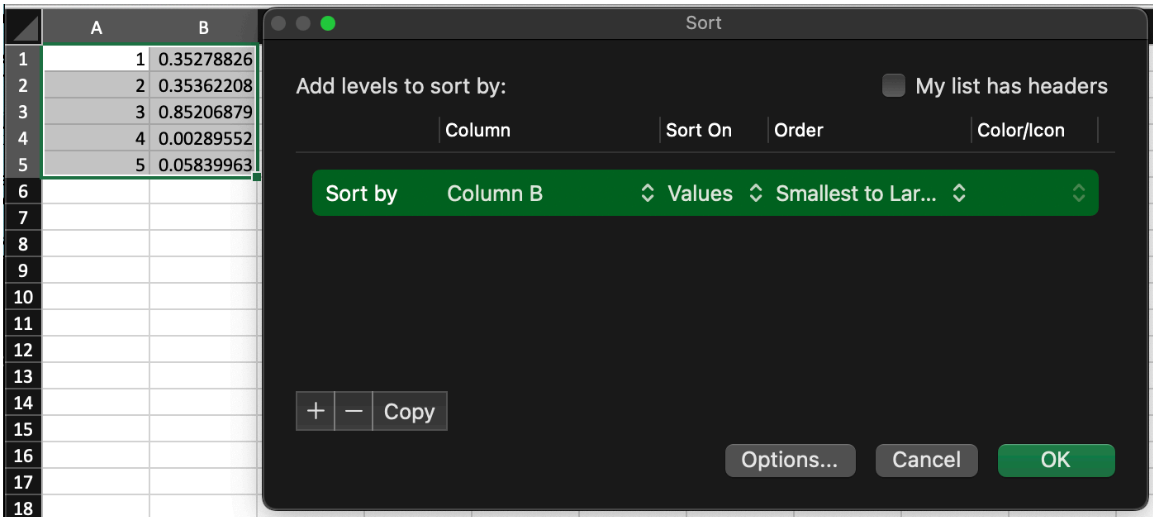

f. Click "OK". This will subsequently randomize the original 1-X number list

	A	B
1	4	0.05339003
2	5	0.14240101
3	1	0.49190007
4	2	0.91766013
5	3	0.18572538

g. **This is the preferred method for large data sets as it eliminates the need to transcribe numbers to excel from random.org**

9.) Using the random generated list created is steps 9 or 10, evaluate each radiograph.

10.) Count the fracture lines visible in the cranial and caudal portions of the keel bone (sternal carina)

11.) To divide the carina into cranial and caudal portions, select the line tool

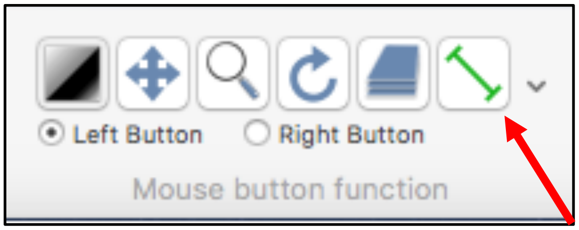

a. Place parallel lines extending from the Carinal apex to the caudal tip (median trabecula) of the carina. Lengths will be generated for these lines.
b. Adjust one of the parallel lines to be exactly half the length of the other, making sure they remain parallel.
c. Place a horizontal line on the lateral surface of the carina at the end point of the previously adjusted line, dividing the carina into equal cranial and caudal portions
d. Delete the parallel lines, leaving the horizontal line for future measurements

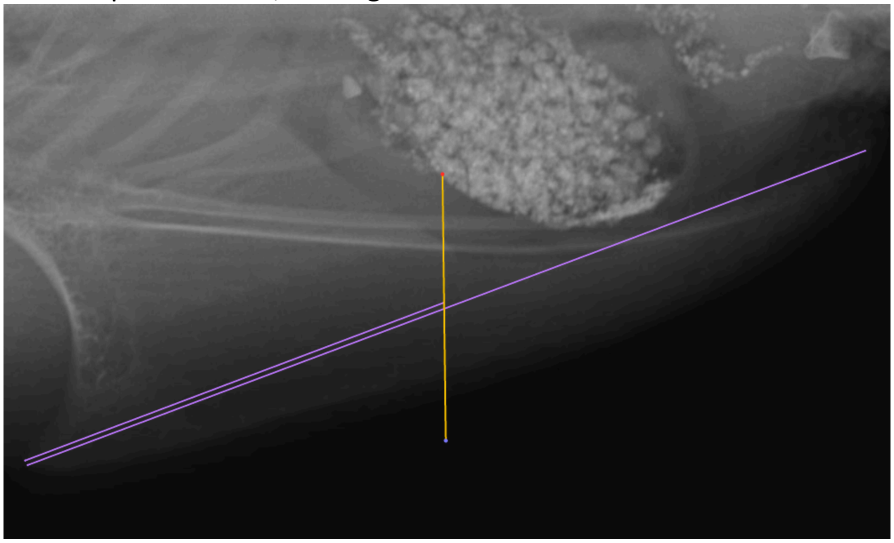

12.) Count the number of cranial and caudal fracture lines (radiolucent lines) and record the totals on the data sheet

13.) Determine if the carina deviates ventrally from the normal shape of the bone on the ventral margin (examples pictured below)

Normal

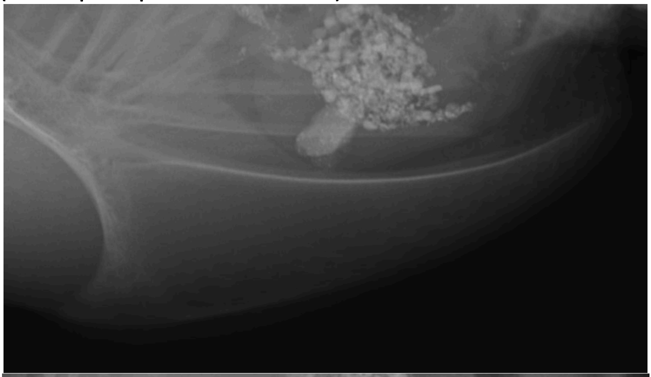

Deviated

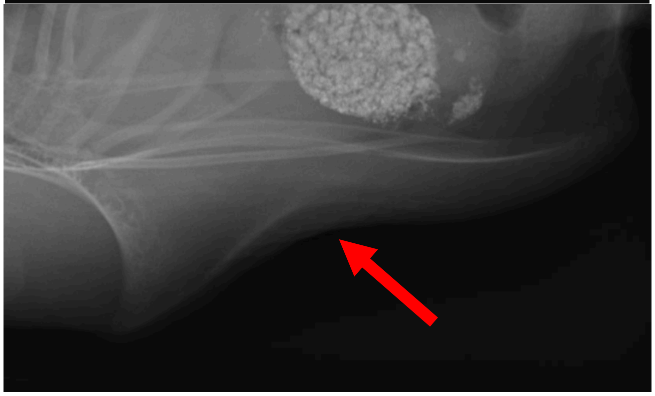

14.) If yes, calculate the proportion of deviated carinal area (POD) (As in Eusemann *et al*. 2018)

a. Using the length tool, place a line on the ventral margin of the carina that lies on the 2 extremes of the bone deviation, making sure the endpoints of the line stop where the deviation transitions back into the normal continuum of the ventral margin. This will simulate a reference ventral margin to be used in later steps
   i. It is understood that the line is a possible underestimation of the total carinal area
b. On both ends of the previously drawn margin, mark an “X” using 2 overlapping lines. The intersection of the lines should be placed on the endpoints of the line drawn in step 14a

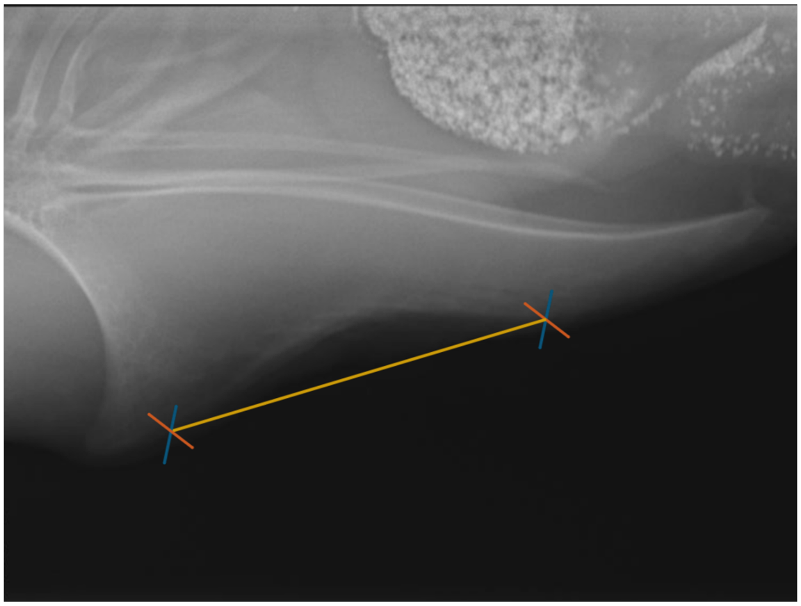

c. Drag the line drawn in step 14a to a different place on the screen as to not obstruct the view of the deviation

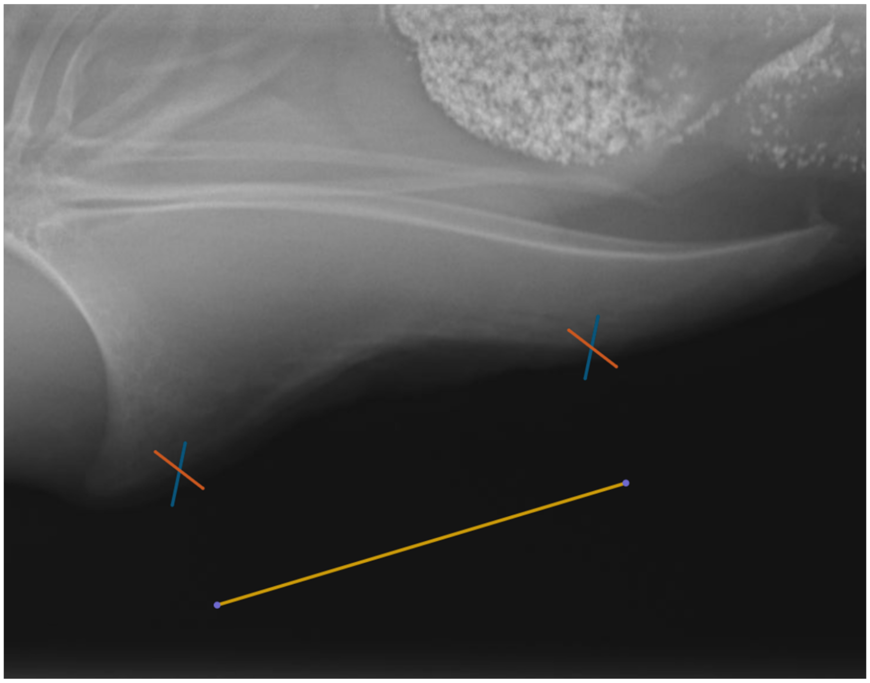

d. Select the pencil tool by clicking the down arrow next to the green length tool
   i. Alternatively, you can press "D" on the keyboard to select the tool

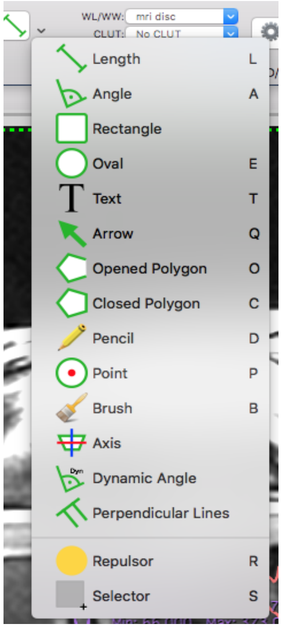

e. Using the pencil tool, outline the ventral deviation of the carina, making sure to start at one “X” and end at the other
f. Release the mouse button and the region of interest will be completed automatically
g. Replace the line drawn in step 14 and add & drag the points of the ROI tracing of the deviation to follow the simulated margin
    i. Dots can be added by double clicking on the region of interest barrier

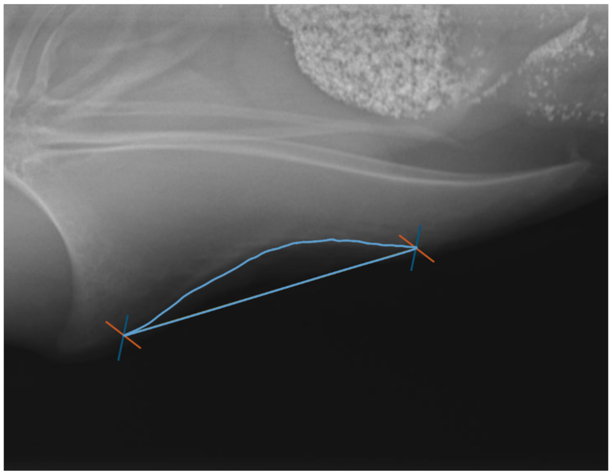

h. Delete the X’s along with the tracing of the deviation as they will not be needed any longer
i. Next, Outline the entire carina, making sure to follow the margin created using the line tool in step 14a

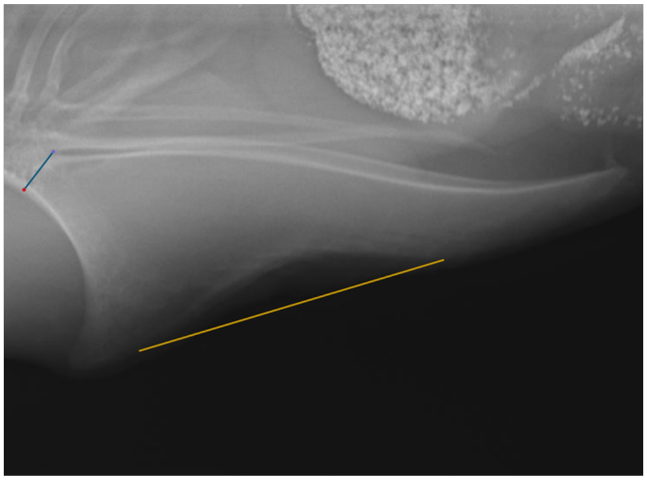

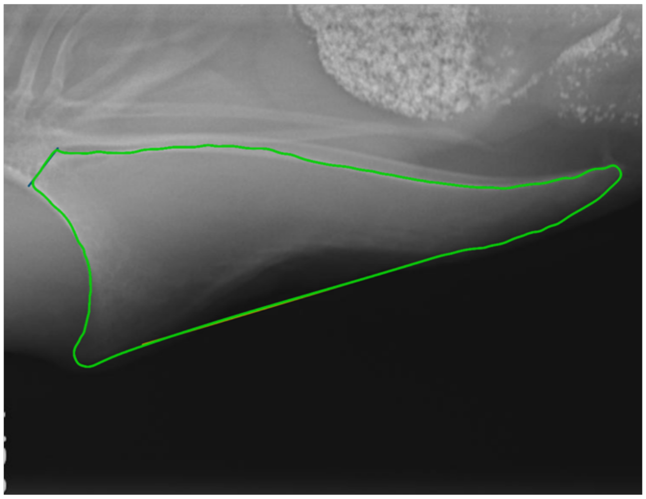

i. When outlining the carina to determine the total carinal area, the craniodorsal border of the carina is determined by placing a line where the body of the sternum fuses with the sternal carina

ii. For ease of following the simulated ventral margin, outline the areas of the carina excluding the line and allow the software to auto-populate the rest of the tracing by releasing the mouse button. Additional points can be placed to alter the tracing afterwards as described in step 14gi

j. The repulsor tool can be used to move any portions of the tracing that are not aligned with the border of the carina

  i. It can be accessed in the tool drop down menu or

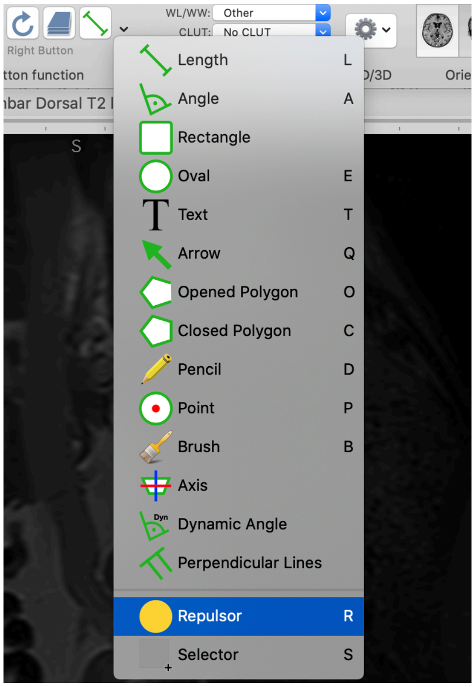

k. Calculate the proportion of the deviation (POD) in the carina and record
    i. (Area of deviation/total carinal area) * 100

15.) Next, identify if there is a change in the angulation of the visceral surface of the carina. If so, then calculate the angle of displacement (AOD)

a. Using the line tool, place a line along the straightest edge of the visceral surface of the carina that is directly preceding the abnormal angulation
b. Place another line on the first identifiable change in the continuity of the visceral surface
c. Using the angle tool, align the rays of the angle with the two lines drawn, and place the vertex at the intersection of the two lines previously drawn

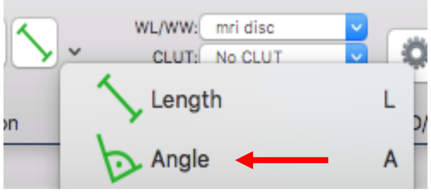

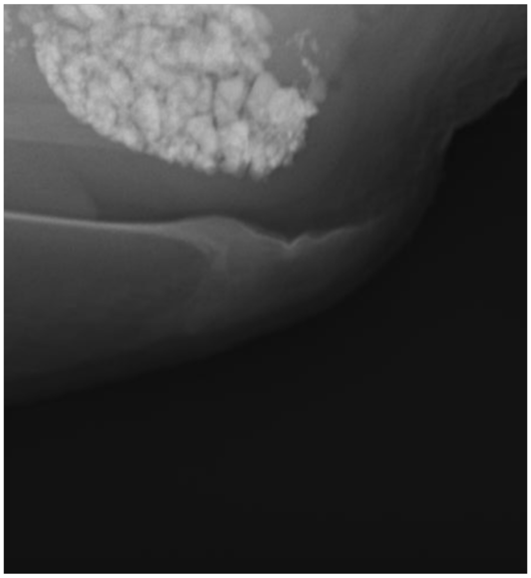

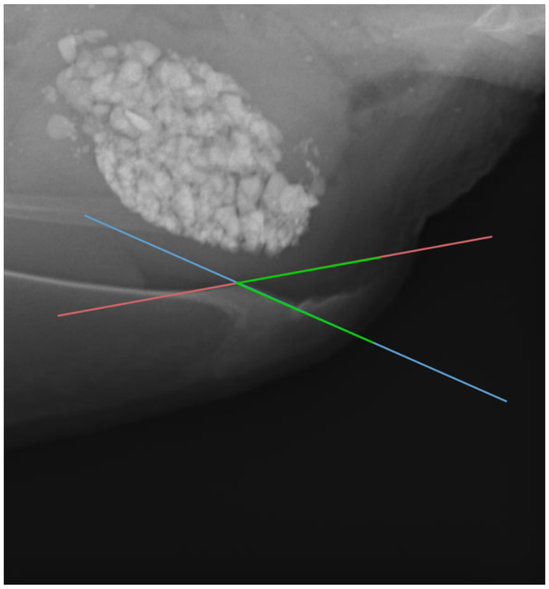

d. Determine whether the angle of displacement is positive (+) or negative (-)
   i. Positive if the angle extends toward the ventral margin of the carina, using the line drawn on the visceral surface of the carina preceding the deformation as a point reference.
   ii. Negative if the angle extends away from the ventral margin of the carina, using the line drawn on the visceral surface of the carina preceding the deformation as a point of reference.

e. Changes in angulation are often accompanied by callus formation as a result of fracture incidence. For calluses that a small and do not interrupt the overall natural curvature of the visceral surface, a determination of the angle of deviation is not necessary (pictured below).

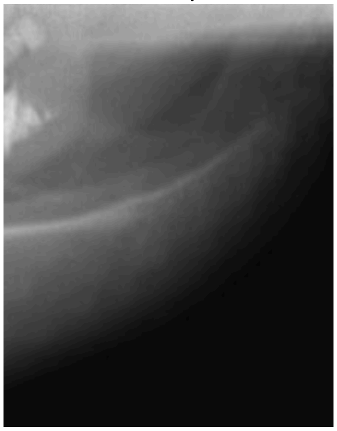

   i. if the callus obstructs the view of the visceral surface, a determination of the angle of deviation is necessary.
   ii. If necessary, refer to the ventral margin of the caudal carina to aid in the determination of whether the carina has an angulation that is not normal.

iii. **Note: The severity of the natural curvature of the caudal carina differs from bird to bird.**

16.) Next, count the number of calluses present on the carina and record.

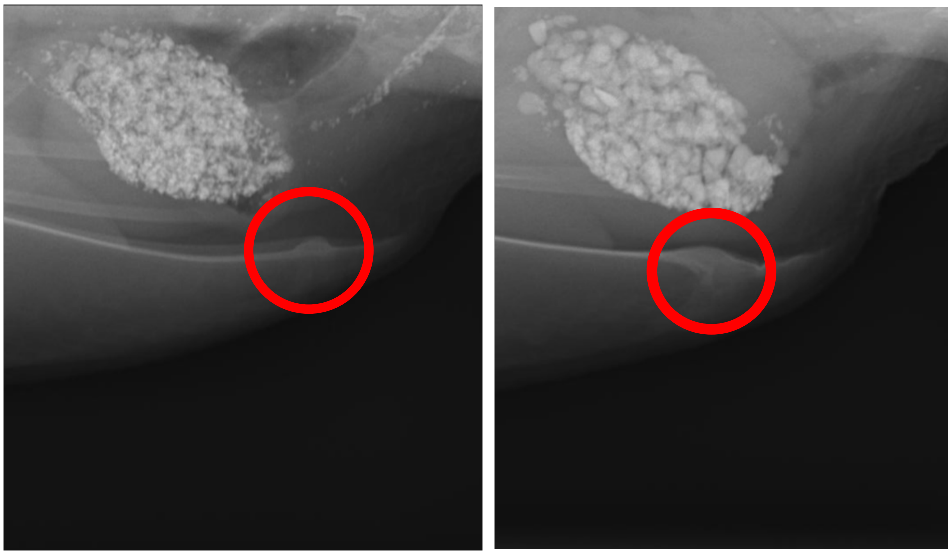

17.) Record triplicate measurements using the previously generated number lists, making sure to complete each replicate before moving on to the next.
