# Supplementary material for: Radiographic, computed tomographic, and histologic characteristics of bone for clinically normal laying hens in a free‐range housing system
Source: Vet Radiol Ultrasound. 2024 Oct 3;66(1):e13443. doi: 10.1111/vru.13443 (PMC11617609; doi:10.1111/vru.13443)
Supplement: Supplementary file 3 — Supporting Information [file VRU-66-0-s004.pdf]

Supplement 2. Detailed, step-by-step protocol for assessing CT keel and tibiotarsal bone and muscle quality in laying hens[a]

1. Enter lab, turn off overhead light and turn on lamp.
2. Log onto the computer with appropriate credentials.
3. Locate Horos image analysis software (v.3.3.6.httpprojecthoros.org/) and open the application.

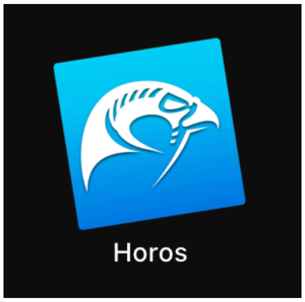

4. The interface, by default, will display a screen containing all files, designated by patient name, patient Id, date acquired etc. Locate the patient name of interest, and select it using the mouse.

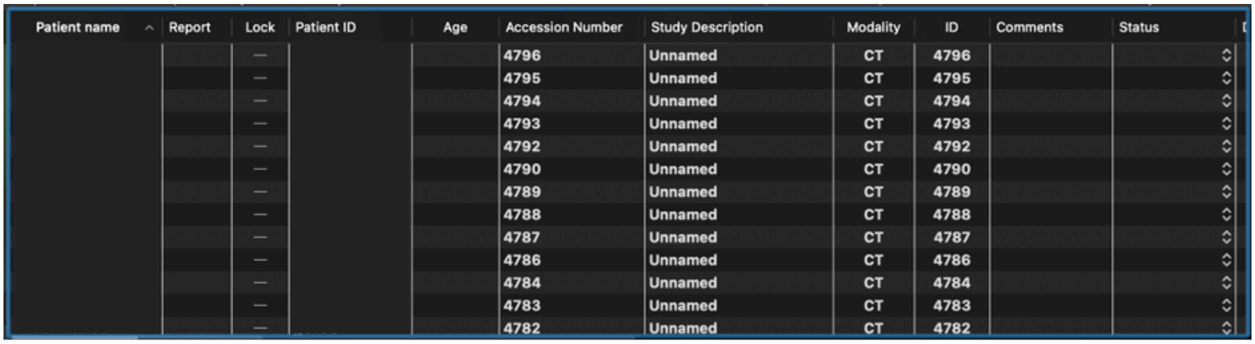

Patient name	Report	Lock	Patient ID	Age	Accession Number	Study Description	Modality	ID	Comments	Status
		—			4796	Unnamed	CT	4796		
		—			4795	Unnamed	CT	4795		
		—			4794	Unnamed	CT	4794		
		—			4793	Unnamed	CT	4793		
		—			4792	Unnamed	CT	4792		
		—			4790	Unnamed	CT	4790		
		—			4789	Unnamed	CT	4789		
		—			4788	Unnamed	CT	4788		
		—			4787	Unnamed	CT	4787		
		—			4786	Unnamed	CT	4786		
		—			4784	Unnamed	CT	4784		
		—			4783	Unnamed	CT	4783		
		—			4782	Unnamed	CT	4782		

5. A subset of reconstruction filters within the patient name will be displayed. Select the "**Head 0.5**" filter for analysis. By default, the images will be displayed in a transverse view.

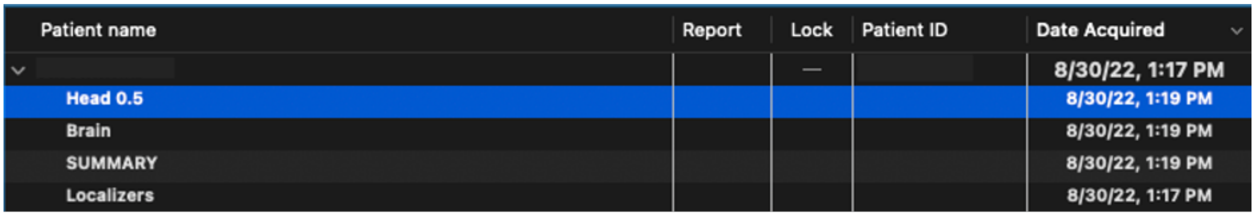

Patient name	Report	Lock	Patient ID	Date Acquired
		—		8/30/22, 1:17 PM
Head 0.5				8/30/22, 1:19 PM
Brain				8/30/22, 1:19 PM
SUMMARY				8/30/22, 1:19 PM
Localizers				8/30/22, 1:17 PM

6. Select "3D Viewer" in the tool bar. From the drop-down menu that is displayed, select "3D MPR"

[a] Reprinted from previous open access publication. Harrison C, Jones J, Bridges W, Anderson G, Ali A, Mercuri J. Associations among computed tomographic measures of bone and muscle quality and biomechanical measures of tibiotarsal bone quality in laying hens. *Am J Vet Res*. 2023;84(11).

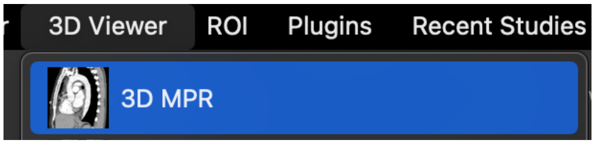

CT Keel Measurements

7. The interface for 3D MPR will display sagittal, transverse, and dorsal view. The set the midline for keel measurements, adjust the crosshairs in the 3 views so the yellow line traverses through the center of the keel in the transverse and dorsal views. In the sagittal view, the blue line should extend from the carinal apex to the median trabecula (caudal tip) of the keel bone (Note: The line in the sagittal view will not overly the carinal apex/median trabecula, but will be offset to the right, parallel to the ventral margin of the keel)

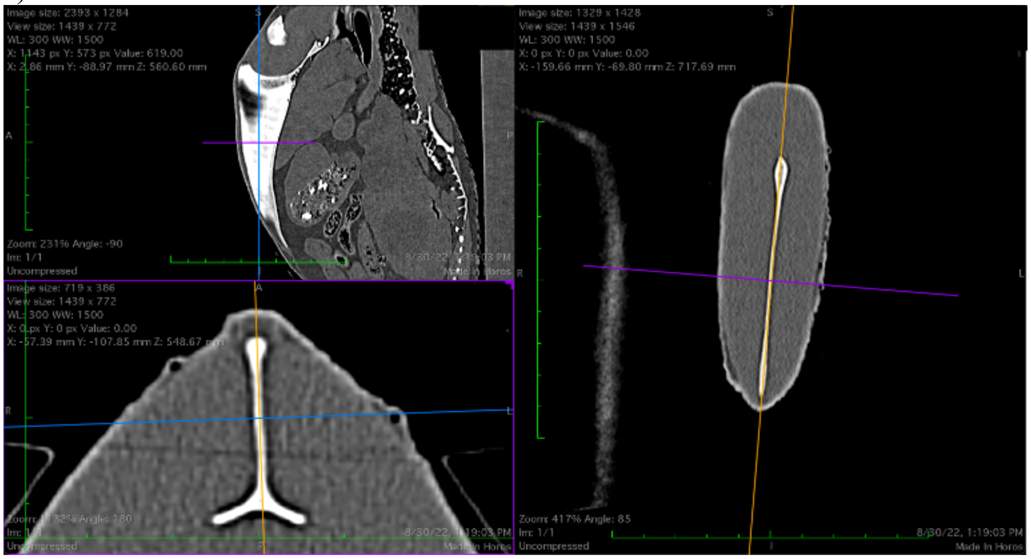

8. Divide the keel bone into proximal, middle, and distal sections
    a. Using the line tool, place a line extending from the carinal apex to the median trabecula. A length will be generated.
    b. Place lines parallel to the line previously drawn exactly ¼ of the length of the original line, making sure that the endpoint of one line coincides with the start of another.
    c. Place horizontal lines at the intersections of the four lines previously drawn, perpendicular to the dorsal margin of the keel bone and parallel to one another.

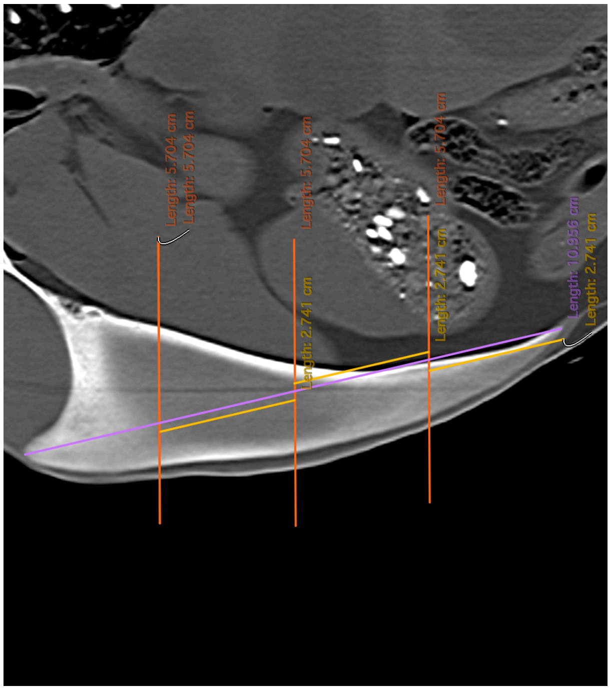

d. Remove all lines except for the three designating the proximal, middle, and distal sections of the keel.

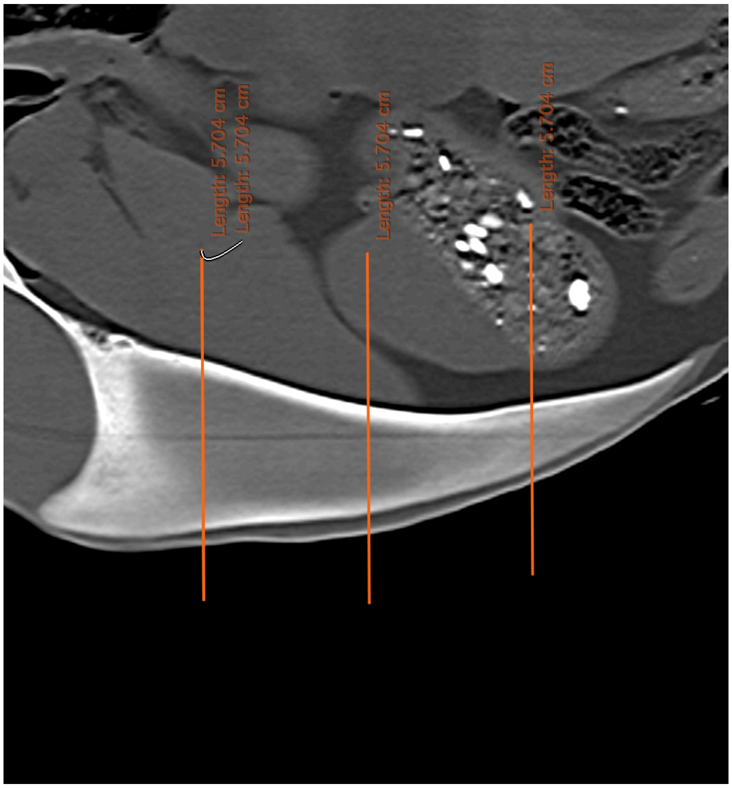

9. Double click the window to display the three views again.
10. Using the left mouse button, select the transverse view by clicking this window once. The active window will be indicated by a purple outline.

11. Using the mouse roller, pan through the slices. You will notice that this simultaneously adjusts the centered location in the sagittal and dorsal views (indicated by the purple line).
12. Align the crosshairs with the first of the three horizontal lines so that the horizontal aspect of crosshairs overlaps the line indicating the proximal location. This will ensure that you are making measurements at that location of the keel.

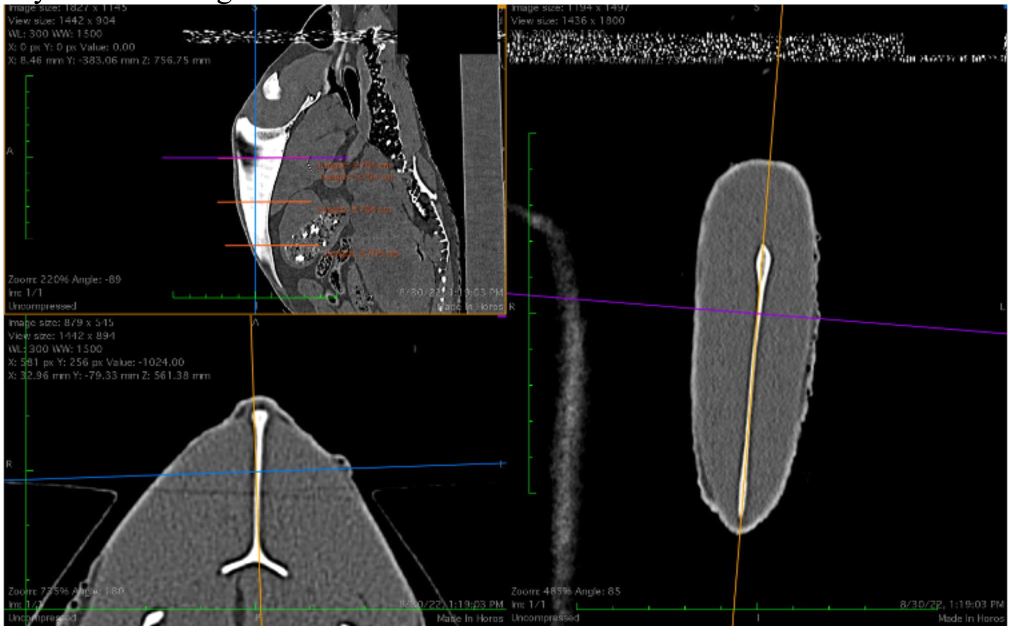

13. Double click the transverse view to maximize the view.
    - **a.** Make sure to refrain from repositioning the image as to not remove the previously drawn lines.

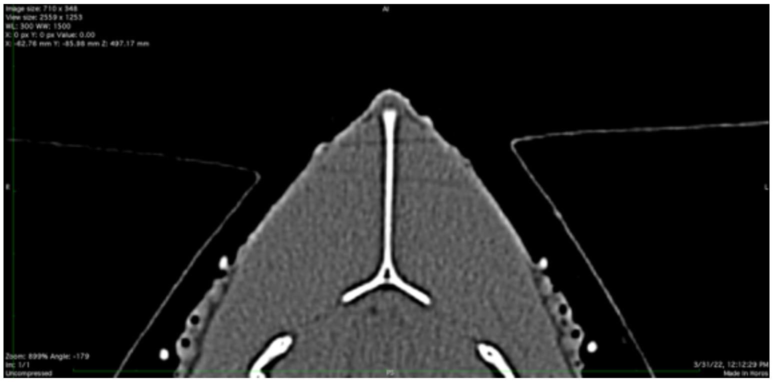

14. Select the downward-facing arrow of the tools section to display a dropdown menu consisting of other software tools. From this menu, select the pencil tool.
    - a. Alternatively, you can press the "D" key on the keyboard to select the pencil tool.
15. With this tool selected, trace the outer margin of the of the cortical bone of the keel, making sure to only zoom in or out on the image to prevent deleting lines indicating separate bone segments.
    - a. When the region of interest tracing is complete, the area, mean, and standard deviation will be displayed. Record these values.

16. Next, trace the inner margin of the keel cortical bone to encompass the medullary cavity.

a. When the region of interest tracing is complete, the area, mean, and standard deviation will be displayed. Record these values.

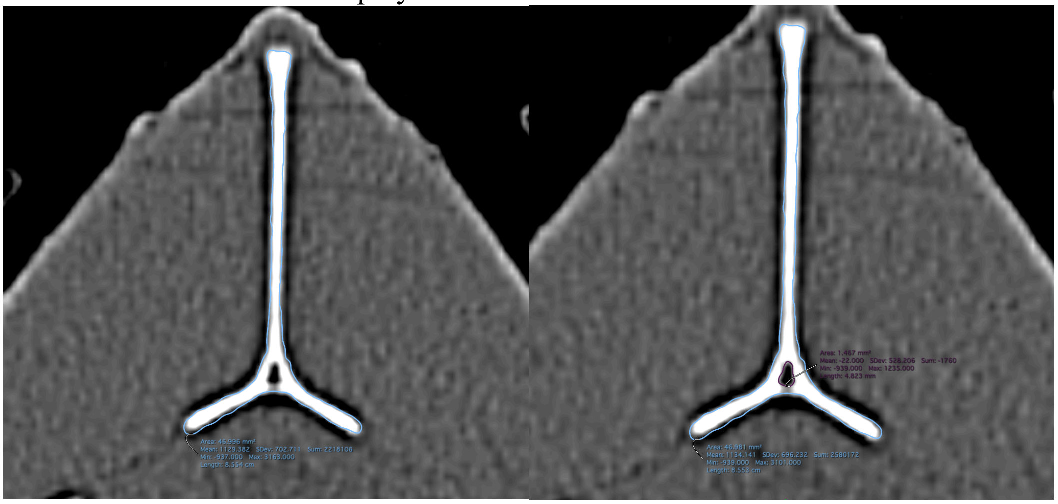

17. Record the cross-sectional area of the muscles surrounding the keel bone.
    a. Using the line tool, place a line horizontal line at the dorsal aspect of the keel
    b. Place a vertical line on the keel passing through both the ventral and dorsal margins and ensure that the lines are perpendicular to one another. Use the angle tool to confirm there is a 90 degree angle between the lines

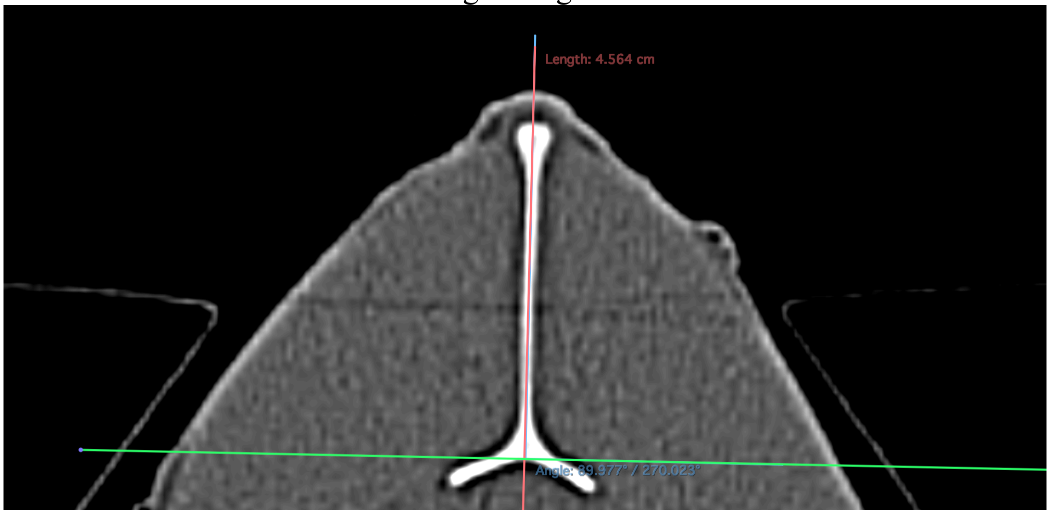

18. Set the WL/WW setting to the abdominal preset and place a region of interest around the muscle mass using the pencil tool. Do not exceed thee horizontal line positioned on the dorsal margin. Record the area.

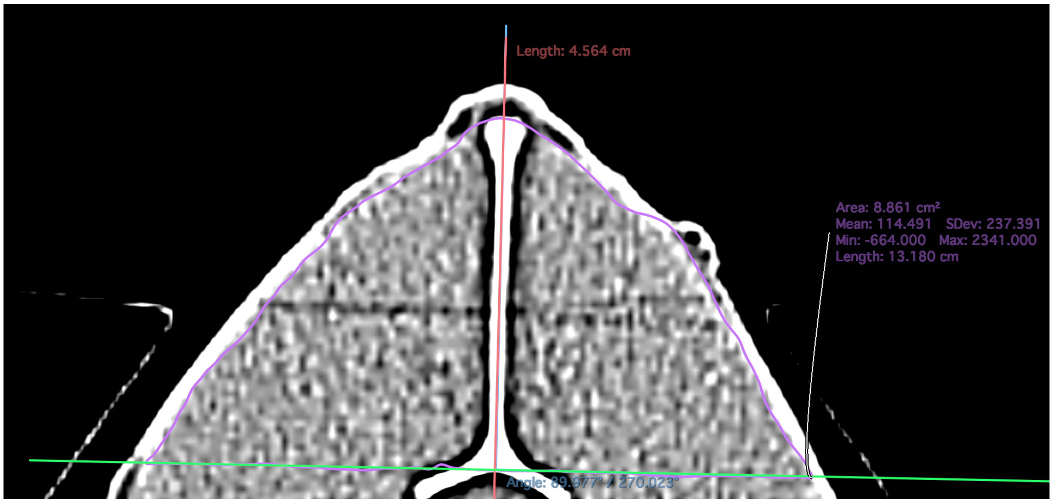

19. Zoom out until you visualize the bone density phantom underneath the back of the bird. Select the oval tool from the tools menu and place circle regions of interest in the three rods of the phantom. Resize the phantom so that it includes the majority of the area of the phantom without extending beyond the margins of the circles.

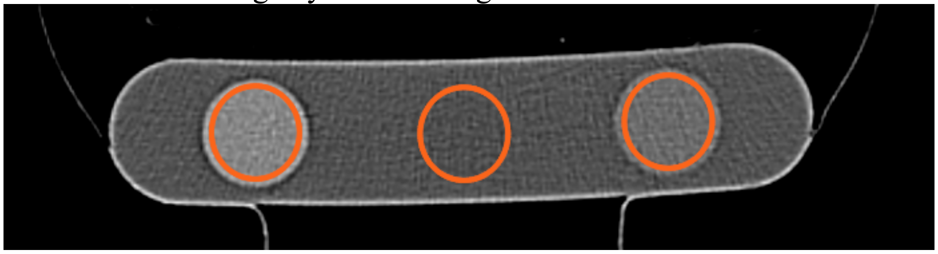

20. Record the mean density and standard deviation values from the phantom. These will be used later to convert the bone CT density values to hydroxyapatite values.
21. Double click the window to return to the display that features the 3 views.
22. With the transverse view selected, using the mouse roller, pan to the second of the three horizontal lines, indicating the middle portion of the keel. Perform the region of interest tracings for the bone and muscle as previously described and record the values.
    a. Record the phantom value as previously done.
23. Pan to the last of the three horizontal lines, indicating the distal segment of the keel. Perform the region of interest tracing as previously described and record the provided values.
    a. Record the phantom value as previously done.

CT Tibiotarsus Measurements

24. Return to the 3MPR window displaying the three views and set the midline for one of the tibiotarsi to ensure than the crosshairs pass through the center of the bone in all three views.
    a. Make sure the CT Bone preset is applied (WW:300/ WL:1500)

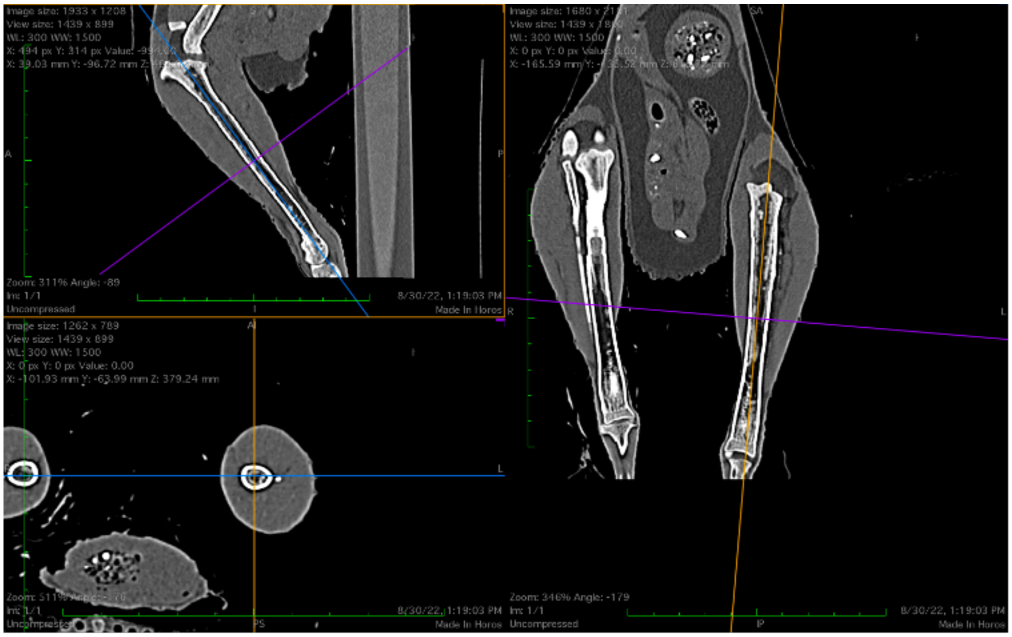

25. Next, maximize the sagittal view of the tibiotarsus and set the proximal, middle, and distal locations using the length tool as done previously for the keel bone in step 8.

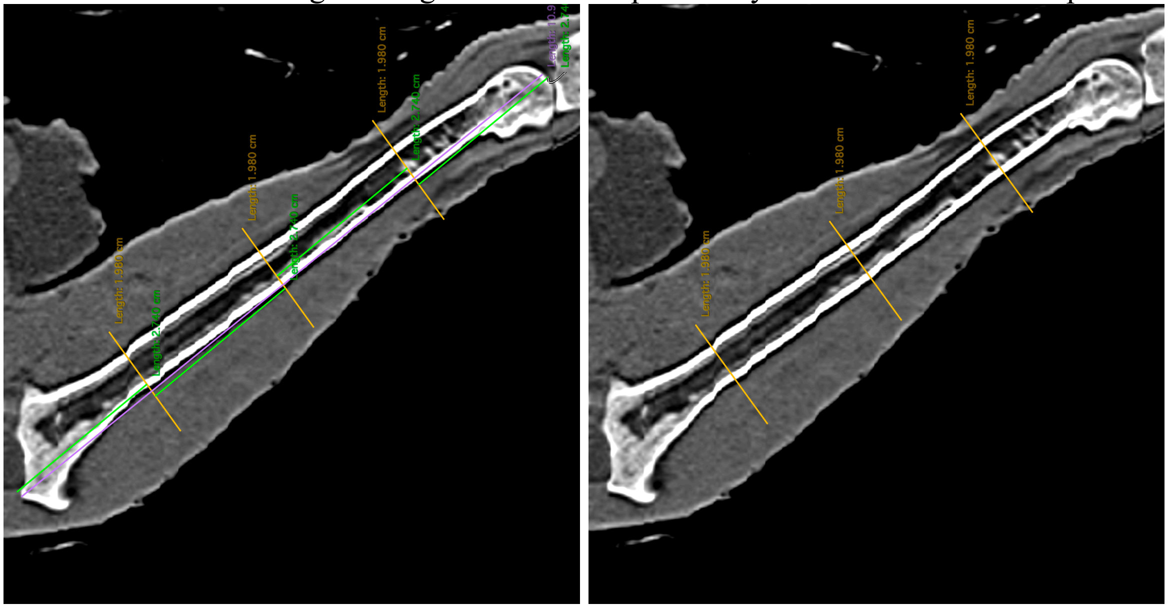

26. Return to the 3-view 3D-MPR window and click in the transverse view to select this window. Using the mouse roller, align the crosshairs with the first of the three lines indicating the proximal, middle and distal locations so that the purple line of the crosshair that is perpendicular to the length of the tibiotarsus overlaps the line indicating the proximal location of the tibiotarsus.

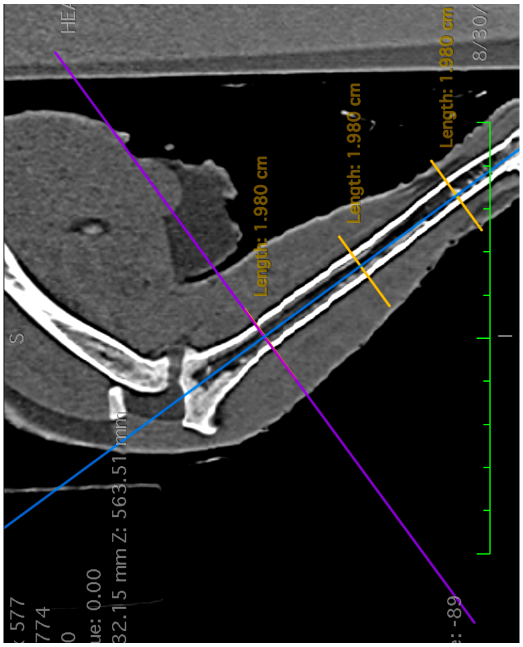

27. Double click the transverse view to maximize this window and place regions of interest around the cortical and medullary bone margins using the pencil tool. Record the area, mean, and standard deviation.

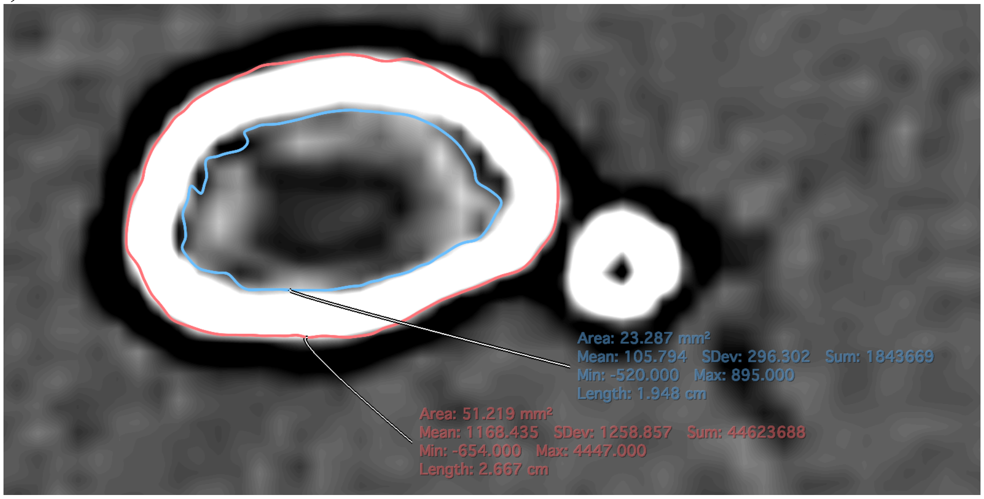

28. Set the WW/WL setting the “abdomen” preset and place a region of interest around the muscle mass surrounding the tibiotarsus. Exclude the skin which will have a higher signal intensity than the muscle.

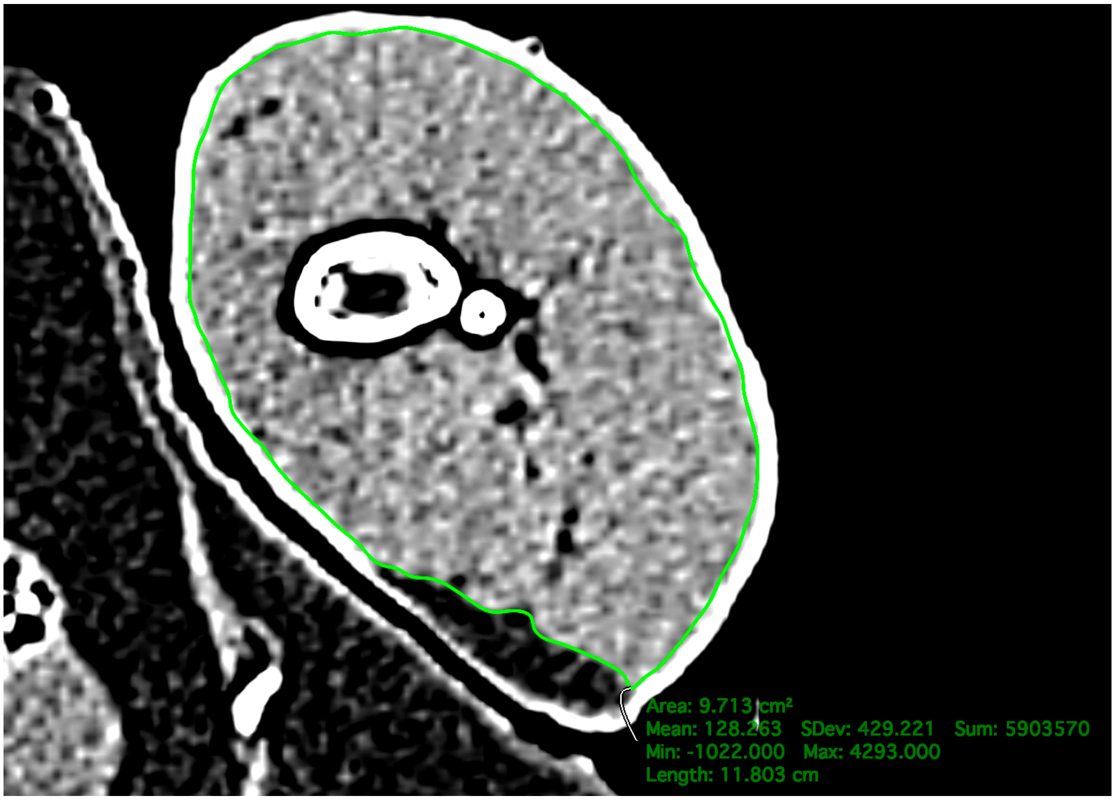

29. Next, perform the hydroxyapatite phantom measurements as previously done for the keel bone in step 19. Record the mean values.

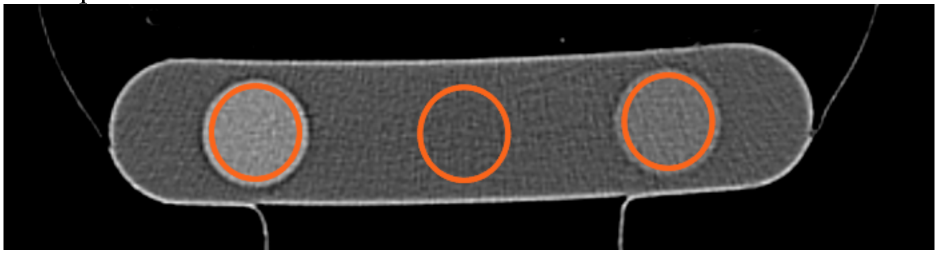

30. Double click in the window to return to 3D-MPR. In the transverse view. Repeat steps 26 – 27 for the middle and distal regions of the tibiotarsus.
31. Next, measure the sagittal cancellous bone density of the distal epiphysis. With the 3D MPR window open, click once in the transverse view to select the window and use the mouse roller to scroll until the distal epiphysis is in view in the sagittal view.

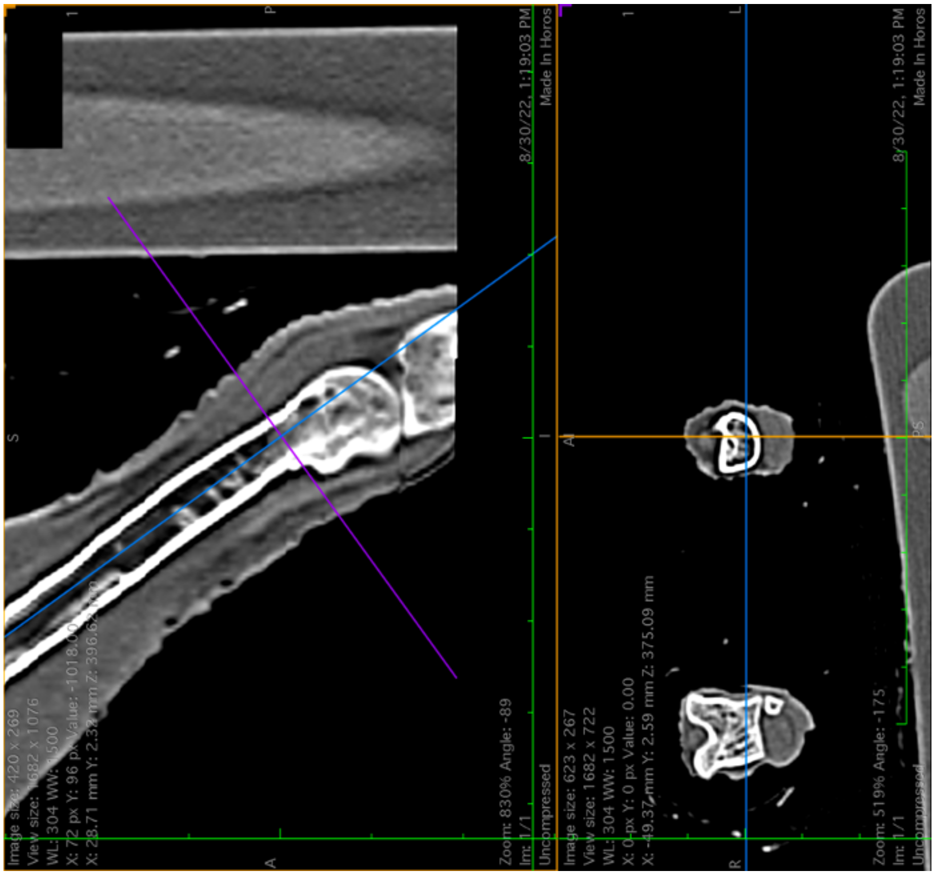

32. Double click the sagittal view to maximize this window and zoom as needed. Place a line perpendicular to the length of the tibiotarsus at the junction of the diaphysis and epiphysis to set the endpoint for the region of interest tracing. Using the pencil tool, outline the epiphysis and record the area, mean, and standard deviation.

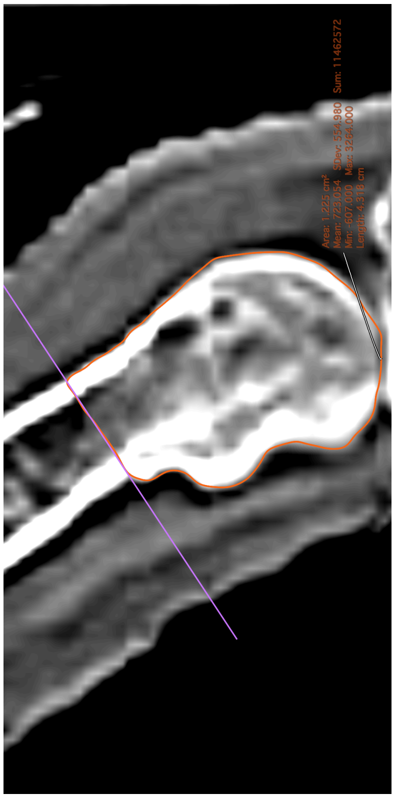

33. Perform steps 24 – 32 for the other tibiotarsus.
