# Supplementary material for: Radiographic, computed tomographic, and histologic characteristics of bone for clinically normal laying hens in a free‐range housing system
Source: Vet Radiol Ultrasound. 2024 Oct 3;66(1):e13443. doi: 10.1111/vru.13443 (PMC11617609; doi:10.1111/vru.13443)
Supplement: Supplementary file 4 — Supporting Information [file VRU-66-0-s003.pdf]

Supplement 3. Results of quantitative radiographic analyses for sternal carina damage in 16 clinically normal, Lowmann Brown laying hens housed in a free-range system

Variable	Range	Median
Number of Cranial Section Complete Fractures	0-1	0
Number of Cranial Section Incomplete Fractures	0	0
Number of Middle Section Complete Fractures	0-1	0
Number of Middle Section Incomplete Fractures	0-2	0
Number of Caudal section Complete Fractures	0-5	2
Number of Caudal Section Incomplete Fractures	0-2	0
Number of Cranial Section Calluses	0-1	0
Number of Middle Section Calluses	0-1	0
Number of Caudal Section Calluses	0-2	0
Proportion of ventral margin deviation (%)	0-17	0

Note: proportion of ventral margin deviation = (area of deviated region/total area of sternal carina) X 100; detailed image analysis protocol provided in Supplement 2
