# Supplementary material for: Radiographic, computed tomographic, and histologic characteristics of bone for clinically normal laying hens in a free‐range housing system
Source: Vet Radiol Ultrasound. 2024 Oct 3;66(1):e13443. doi: 10.1111/vru.13443 (PMC11617609; doi:10.1111/vru.13443)
Supplement: Supplementary file 5 — Supporting Information [file VRU-66-0-s001.pdf]

Supplement 4: Results of quantitative computed tomographic analyses for four, clinically normal Lohmann Brown laying hens housed in a free-range system.

**Anatomic region**	**Tibiotarsus**									**Sternum**	
**Variable category**	**Total bone**		**Cortical bone**		**Muscle**			**Cancellous bone distal**			
**Variable**	**Area (mm^2)**	**BMD (mg/cm^3 [CaHA])**	**Area (mm^2)**	**BMD (mg/cm^3 [CaHA])**	**Area (cm^2)**	**Corrected mean density (hu)**		**Area (mm^2)**	**BMD (mg/cm^3 [CaHA])**	**Ossification (%)**	**Lateral angulation of carina (degrees)**
**Range proximal**	55.04 to 66.57	672.24 to 750.16	27.33 to 31.84	355.98 to 558.47	9.95 to 11.24	99.00 to 119.98	**Range**	1.21 to 1.43	253.01 to 346.92	100 to 100	0 to 28.37
**Median proximal**	55.7	705.3	28.5	498.2	10.5	104.1	**Median**	1.3	305.4	100.0	22.9
**Range middle**	39.18 to 48.70	796.26 to 951.76	23.22 to 27.11	505.70 to 754.15	4.82 to 16.74	130.34 to 411.80					
**Median middle**	43.2	877.5	25.0	603.7	5.3	138.2					
**Range distal**	39.04 to 47.82	692.99 to 788.95	22.11 to 25.93	602.36 to 831.94	1.23 to 1.70	252.64 to 337.38					
**Median distal**	43.0	741.6	23.4	790.6	1.4	291.6					

Notes: BMD, bone mineral density; hu, CaHA, calcium hydroxyapatite; Hounsfield units; corrected mean density = muscle hu – water phantom hu; ossification % = (mid-sagittal area of ossified sternum/mid-sagittal area of total sternum) X 100; area of proximal tibiotarsus cancellous bone calculated from mid-sagittal planar image. Detailed image analysis protocol provided in Supplement 3.
